# CCR2^+^ Macrophages Promote Orthodontic Tooth Movement and Alveolar Bone Remodeling

**DOI:** 10.3389/fimmu.2022.835986

**Published:** 2022-02-04

**Authors:** Hao Xu, Shuting Zhang, Adwait Amod Sathe, Zhichun Jin, Jiani Guan, Wen Sun, Chao Xing, Hanwen Zhang, Bin Yan

**Affiliations:** ^1^ Department of Orthodontics, The Affiliated Stomatological Hospital of Nanjing Medical University, Nanjing, China; ^2^ Jiangsu Key Laboratory of Oral Diseases, Nanjing Medical University, Nanjing, China; ^3^ Jiangsu Province Engineering Research Center of Stomatological Translational Medicine, Nanjing, China; ^4^ School of Biomedical Engineering and Informatics, Nanjing Medical University, Nanjing, China; ^5^ Eugene McDermott Center for Human Growth and Development, University of Texas Southwestern Medical Center, Dallas, TX, United States; ^6^ Department of Bioinformatics, University of Texas Southwestern Medical Center, Dallas, TX, United States; ^7^ Department of Population and Data Sciences, University of Texas Southwestern Medical Center, Dallas, TX, United States; ^8^ School of Basic Medical Sciences, Nanjing Medical University, Nanjing, China; ^9^ Key Laboratory of Targeted Intervention of Cardiovascular Disease, Collaborative Innovation Center for Cardiovascular Disease Translational Medicine, Nanjing Medical University, Nanjing, China

**Keywords:** single-cell sequencing, macrophage, bone remodeling, inflammatory, CCR2

## Abstract

During mechanical force-induced alveolar bone remodeling, macrophage-mediated local inflammation plays a critical role. Yet, the detailed heterogeneity of macrophages is still unknown. Single-cell RNA sequencing was used to study the transcriptome heterogeneity of macrophages during alveolar bone remodeling. We identified macrophage subclusters with specific gene expression profiles and functions. CellChat and trajectory analysis revealed a central role of the *Ccr2* cluster during development, with the CCL signaling pathway playing a crucial role. We further demonstrated that the *Ccr2* cluster modulated bone remodeling associated inflammation through an NF-κB dependent pathway. Blocking CCR2 could significantly reduce the Orthodontic tooth movement (OTM) progression. In addition, we confirmed the variation of CCR2^+^ macrophages in human periodontal tissues. Our findings reveal that mechanical force-induced functional shift of the *Ccr2* macrophages cluster mediated by NF-κB pathway, leading to a pro-inflammatory response and bone remodeling. This macrophage cluster may represent a potential target for the manipulation of OTM.

## Introduction

Orthodontic treatment is a safe and effective option to correct malocclusion of varying classes with the significant limitation of long therapy time. Orthodontic tooth movement (OTM) is achieved by remodeling alveolar bone in response to mechanical force loading, which causes bone resorption on the pressure side and bone formation on the tension side (1). During bone remodeling, local inflammation could induce bone reconstruction through various stromal cells in response to mechanical force and increased damage-associated molecular patterns (DAMPs). Our previous study indicated that macrophages play a crucial role in regulating bone remodeling during OTM (2).

With a highly flexible nature and the ability to rapidly adapt to the local microenvironment, macrophages play an essential role during bone modeling and remodeling by serving as progenitors of osteoclasts, modulators of inflammation, and effectors of mechanical force (3). Previous studies show that as an essential source of pro-inflammatory cytokines including IL-1β, IL-6, TNF-α and GM-CSF both locally and systemically, macrophages are crucial during inflammation-mediated bone remodeling (4). Macrophages may therefore become a potential target in modulating bone remodeling progression. Recent studies show that M1-like macrophage polarization promotes alveolar bone resorption and consequent OTM after applying mechanical force (5). However, the origin, function, and dynamic changes of macrophages during mechanical force-induced alveolar bone remodeling remain elusive.

The origin of macrophages in periodontal tissues is crucial to modulate macrophage functions in bone remodeling during OTM. Previous studies have demonstrated that macrophages are derived from embryonic (yolk sac, fetal liver) and adult (bone marrow) precursor cells under physiological and disease conditions such as myocardial infarction (6). However, the origin of macrophages in OTM bone remodeling is unclear. Moreover, the signature genes and functions of bone resident macrophages (“OsteoMacs”) have not been fully defined thus far (7, 8). Considering the critical role of macrophages in OTM, we sought to investigate the clustering, origins, and function of macrophages using single-cell RNA sequencing (scRNA-seq). Single-cell RNA sequencing allows us to analyze individual cell heterogeneity at the transcriptome level and explore the contributions of different cell subtypes at steady-state and under pathological conditions. A recent study constructed a single-cell atlas of mandibular alveolar bone stromal cells. It revealed that macrophages are the most prominent cell population that interacts with mesenchymal stem cells (MSCs) through oncostatin M (OSM) (9). Due to the complicated heterogeneity of macrophages, the combined use of FACS and scRNA-seq when parsing alveolar bone macrophages is needed.

Here, we compared the heterogeneity of macrophages in murine alveolar bone before and 7 days after OTM through scRNA-seq. We found 12 clusters in the control group and 8 clusters in the OTM group, with drastic functional and clustering switches during OTM. We detected the changes of major macrophage clusters in human periodontal tissues. Most importantly, we identified the *Ccr2* cluster, which exhibited the most decisive functional and intercellular communication switch during OTM. Trajectory analysis indicated that the proliferative differentiation stages of macrophages originated from the *MHC-II* cluster. After OTM, the number of macrophages increased in the proliferative state and decreased in the basal state, suggesting that macrophages were activated. Furthermore, we confirmed the pro-OTM function of the *Ccr2* cluster through a specific inhibitor of CCR2, RS504393. In summary, our study reveals novel macrophage diversity during OTM, which may provide precise therapeutic targets for modulation of the inflammatory microenvironment in future orthodontic clinical treatment.

## Materials and Methods

### Animal Models

Adult C57/B6 J mice (male, 25–28 g, 8 weeks old) were used in this study. All animals were maintained in a virus- and parasite-free barrier facility and exposed to a 12-h/12-h light/dark cycle under standard conditions in the Medical Experimental Animal Center of Nanjing Medical University, China. All study protocols were approved by the Committee of Nanjing Medical University for Animal Resources.

### Application of Orthodontic Devices

20 mice were randomly divided into an OTM group and a control group for scRNA-seq, 10 mice were randomly divided into an OTM group and an OTM with CCR2 inhibitor RS-504393 intraperitoneal injection group. The mechanical force was applied mice as previously described (10), with modification. In the OTM group, the left first molars were ligated to the maxillary incisors using a NiTi coil spring, with a force of approximately 30 g to induce mesial movement. The appliances were checked without anesthesia every 24 h and reinstalled immediately if they fell off. Here, the NiTi coil didn’t fall off and we didn’t reinstall the appliance. The mice were fed a soft diet to relieve discomfort and sacrificed after 7 days of the force application.

### Drug Treatment

For RS504393 treatment, 5mg/kg RS504393 (HY-15418; MCE) or vehicle was intraperitoneal injected in mice daily after orthodontic devices application (11).

### Tissue Collection and Single-Cell Sorting

At the designated termination time points, animals were euthanized by carbon dioxide overdose. After extraction of the left maxillary first molar, the alveolar bone tissue was immediately collected and washed with ice-cold PBS with 1% FBS. All cells from alveolar bones were obtained by digestion with 3 mg/mL type I collagenase (Sigma‐Aldrich) for 60 min at 37°C, and single-cell suspensions were obtained *via* passage through 70-µm cell strainers (Solarbio). Macrophages were isolated through gradient centrifugation and then incubated on ice for 30 min using fluorescently conjugated antibodies against F4/80 (PerCP-Cyanine 5.5-conjugated; eBioscience) and CD11b (APC-conjugated; eBioscience). Then, sorting was performed using FACS (FACSAria Fusion, BD). The forward-scatter area (FCS-A) versus side-scatter area (SSC-A) was used to gate out damaged cells. Live cells were stained with 1 µl of Hoechst blue, and dead cells without staining were gated out. Cells stained with anti-F4/80-PerCP-Cy5.5 were collected in a medium containing BSA.

### Single-Cell RNA Sequencing Library Construction Using the 10x Genomics Platform

Samples were washed twice in PBS (Life Technologies) plus 0.04% BSA (Sigma‐Aldrich). Each wash was performed with a 5-min centrifugation at 330 g and resuspension in 1 ml PBS plus 0.04% BSA. Sample viability was assessed using trypan blue (Thermo Fisher) and a hemocytometer (Thermo Fisher), and the appropriate volume for each sample was calculated. After droplet generation (the generation of GEMs with RT primers), samples were transferred onto a prechilled 96-well plate (Eppendorf), and reverse transcription was performed. Next, post GEM-RT was recovered using Recovery Agent provided by 10x Genomics followed by DynaBead cleanup. Purified cDNA was amplified and cleaned up using SPRIselect reagent (Beckman). Samples were taken (1μL) and run on a Bioanalyzer High Sensitivity Chip (Agilent Technologies) to determine cDNA concentration. cDNA libraries were prepared as outlined by the Single Cell 3′ Reagent kit v2 user guide with appropriate modifications to the PCR cycles based on the calculated cDNA concentration (as recommended by 10x Genomics). The completed library contains the complete Illumina P5 and P7 terminals. The 1-16 bp segment of Read 1 was the 10x barcode, the 17-26 bp segment was the UMI, and the i7 index read was the sample index. Samples were sequenced on a HiSeq 2500 with the following run parameters: read 1, 26 cycles; read 2, 98 cycles; and i7 index, 8 cycles.

### Single-Sell RNA Sequencing Analysis

We used Cell Ranger version 3.0.0 (10x Genomics) to process the raw sequencing data. Raw BCL files were converted to FASTQ files and aligned to the mouse mm10 reference transcriptome. The transcript counts of each cell were quantified using barcoded UMI and 10xBC sequences. The gene x cell expression matrices were loaded into the R package Seurat version 3.0.0 for downstream analyses. Cells with low quality were filtered out based on < 200 genes being detected per 1 000 UMIs and mitochondrial gene content > 5%. Only genes found in more than 3 cells were retained. “LogNormalize”, the Seurat default global-scaling normalization method, was performed. In this method, UMI counts are first scaled by the total sequencing depth (‘size factors’) followed by pseudocount addition and log-transformation.

With the above filters in place, we obtained 12 753 genes from the control sample and 12 257 genes from OTM sample. The highly variable features (genes) were then calculated with “FindVariableFeatures” in Seurat, which uses a mean variability plot. Here, the average expression and dispersion per feature were calculated, and features were divided into bins to obtain z-scores for dispersion per bin.

After regressing out the number of UMIs and the percentage of mitochondrial gene content, the resultant data were scaled, and dimensional reduction was performed with principal component analysis (PCA) and visualization using UMAP plots. The number of principal components (n=12 and 8 for control and OTM, respectively) to use in the downstream analysis was calculated based on a jackstraw and elbow plot of the same data.

For each sample, a shared nearest neighbor (SNN) graph was constructed with “FindNeighbors” in Seurat by determining the k-nearest neighbors of each cell. The clusters were then identified by optimizing this SNN modularity using the “FindClusters” function. This allowed for the sensitive detection of rare cell types. We obtained 12 and 8 clusters for the control and OTM, respectively, with a resolution of 0.6.

Differential expression testing was carried out using the Wilcoxon rank sum test in Seurat. This strategy was carried out to obtain the top markers for each cluster. The genes identified as relatively overexpressed in a cluster compared to all other cells in a sample were termed markers.

The cells identified in each sample showed 4 cell types in common, namely, *Ccr2, Mmp8, MHC-II* and *Mki67*, and others that were unique to each sample. To obtain a deeper understanding of the differences between the two samples, differential expression analysis was also carried out after combining the two samples using the method described by Tim et al. where canonical correlation analysis was applied to identify correspondences between samples and create a common reference (12). Here, the analysis was carried out between cells belonging to the same cluster (cell types) as defined in each sample (*Ccr2, Mmp8, MHC-II and Mki67*) and the cells unique to each sample. Differential expression was also carried out between cells belonging to the same cell type but from different sample types. Specific marker distributions in clusters were represented as feature plots, violin plots, and heatmaps using the Seurat tool.

### Functional Enrichment Analysis

The top 60-65 markers (adj. p-value < 0.05) per cluster were used to identify the functional enrichment categories using Metascape. We used pathway gene sets from the biological processes of Gene Ontology (http://www.geneontology.org/), Kyoto Encyclopedia of Genes and Genomes (KEGG), and Reactome (http://www.reactome.org/).

Gene set enrichment analysis (GSEA) was performed using the Canonical pathways in curated gene sets and 50 hallmark gene sets in the MSigDB databases to identify the pathways that were induced or repressed in *Ccr2* clusters between the control and OTM groups by the GSEA software (13).

### Single-Cell Trajectory Analysis

Single-cell trajectory analysis with reversed graph embedding was performed following the methods implemented by Dick et al (14), which was designed to tease out any cell fate decisions that could be ascertained by gene regulation. The output from Seurat were fed into the Dynverse tool, and dimensionality reduction was applied using the parameters of 2 and 3 for maximum components and number of dimensions, respectively. The trajectory was built by ordering the cells on a pseudotime based on the differentially expressed genes obtained in the previous Seurat analysis. The *MHC-II* cluster was used as the root state. In both samples, we identified 2 branches defined by the gene expression profiles, as shown in the trajectory figures generated by the “plot_cell_trajectory” function. The figures were generated per cluster for each sample to highlight the different cell fate decisions leading to the two branches in the figures. These figures were also generated for the individual markers and genes changing as a function of pseudotime. All pseudotime-dependent genes were visualized using a heatmap reflecting the kinetic trends of these genes.

### Cell-Cell Interaction Analysis

CellChat was used to perform cell-cell communication calculation and analysis for the macrophage clusters (15). The CellChat results were used to reveal both incoming communication patterns of target cells and outgoing communication patterns of the secreting cells. To reveal the strength of specific pathways among macrophage clusters, we selected the most prevalent CCL signaling pathway for further visualization.

### Microcomputed Tomography Scanning and Analysis

All maxillary samples were separated and scanned using a microcomputed tomography (micro-CT) machine (Skyscan 1176, Bruker) with a standard acquisition protocol (50 kV, 500 μm, and 0.3 mm voxel size). A single-blinded rater renamed the micro-CT data with randomly generated codes, and the files were imported into InVivoDental for reconstruction. *Via* 3D reconstruction, each first and second molar was layered buccally up to the midpoint section of the marginal ridges, where distal pits were visible to be marked. The distance between the distal pit of the first molar and that of the second molar was recorded to measure tooth movement.

### Cell Culture and Reagents

Bone marrow–derived macrophages (BMDMs) were isolated and cultured as previously described (16). Briefly, mouse bone marrow cells were flushed out from the bone marrow cavity of femurs and tibias. The cells were then washed, resuspended, and seeded into culture plates. These cells were cultured for 7 days in DMEM supplemented with 10% FBS, antibiotics (100 U/mL penicillin and 100 mg/mL streptomycin; Gibco), and 25% (v/v) L929-conditioned medium. The supernatant of a murine fibroblastic cell line, L929, was collected as the source of M-CSF/CSF-1.

The BMDMs were pretreated with RS-504393 (1 µg/ml, HY-15418; MCE) for 30 min. Then, BMDMs were induced to differentiate into pro-inflammatory macrophages with LPS (100 ng/mL, 055:B5; Sigma-Aldrich) or into restorative macrophages with IL-4 (20 ng/mL, 214-14; PeproTech) for 24 hours. BMDMs were pretreated with P65 inhibitor Helenalin (10μm, HY-119970; MCE) for 2 hours, then treated with CCL2 (100ng/ml, 479-JE; R&D) for 3 hours.

Loss-of-function analysis was performed using siRNAs targeting CCR2 purchased from GenePharm. The cells were seeded in 6-well plates and transfected with 100 nM siRNA using Lipofectamine 2000 (Invitrogen) according to the manufacturer’s instructions. The knockdown of each target gene was confirmed by qRT-PCR. The cells were used for subsequent experiments 48 h after transfection.

### Immunofluorescence Staining

For the immunofluorescence staining, fresh human periodontal tissues were obtained by means of curettage with scalpel blade from patients who need to extract the maxillary first premolars for orthodontic treatment in Department of Orthodontics, the Affiliated Stomatological Hospital of Nanjing Medical University. We first extracted the first premolar on one side (control group), and then extracted another first premolar on the other side 7 days after the force was applied. The study was approved by the Research Committee of the Affiliated Stomatological Hospital of Nanjing Medical University (PJ2021-058-01). After fixation with 4% paraformaldehyde, the samples were dehydrated, and embedded in Tissue-Tek O.C.T. (SAKURA) to surround and cover the tissue specimens. The specimens were sliced into 6-µm-thick sections and washed with PBS for 30 min. Subsequently, samples were blocked for 30min with 10% BSA at room temperature and incubated with anti-CD68 (1:200, ab955; Abcam) and CCR2 antibodies (1:100, bs-0562R; Bioss) overnight at 4°C to detect CCR2^+^ macrophages. Sections were then incubated with fluorescein Cy3-conjugated secondary antibody (1:200, A0521; Beyotime) and fluorescein CoraLite488-conjugated secondary antibody (1:200, SA00013-2; Proteintech) for 1.5 hours at room temperature. Nuclei were counterstained with 4′,6-diamidino-2-phenylindole (DAPI).

The cells were fixed in 4% formaldehyde diluted in PBS for 15 min, permeabilized with 0.2% Triton X-100, treated with blocking buffer (10% BSA in PBS), and then incubated overnight with the primary antibody against iNOS (1:500, ab15323; Abcam) or p65 (1:500, ab32536; Abcam) at 4°C overnight. The cells were then incubated with a fluorescein Cy3-conjugated secondary antibody (1:200, A0507; Beyotime) or a fluorescein CoraLite488-conjugated secondary antibody (1:200, SA00013-2; Proteintech) for 1.5 hours at room temperature.

### RNA Preparation and Quantitative Real-Time Polymerase Chain Reaction (qRT-PCR)

Total RNA was isolated from cultured macrophages after various treatments and from mouse alveolar bones using TRIzol reagent (Life Technologies, CA, USA) according to the manufacturer’s instructions. Complementary DNA was amplified from 2 μg of total RNA in a volume of 10 μL using PrimeScript Reverse Transcription Master Mix (TaKaRa). Quantitative real-time PCR was performed using SYBR Green Premix Ex Taq (TaKaRa) on an ABI 7900 system (Applied Biosystems, USA). All the primer sequences are listed in **Supplementary Table 2**. All data were normalized to *Gapdh* expression. Quantification of qPCR results was performed by the 2^-△CT^ method.

### Cell Lysis and Western Blotting

Macrophages were lysed with radioimmunoprecipitation assay (RIPA) buffer (Santa Cruz) for protein extraction. Then, sodium dodecyl sulfate–polyacrylamide gel electrophoresis (SDS-PAGE, 10%) was performed to separate the proteins, which were then transferred to PVDF membranes (Millipore). The membranes were incubated overnight with primary antibodies against phospho-p65 (1:1000, #93H1; CST), p65 (1:1000, #8242; CST), CCR2 (1:1000, ab203128; Abcam), or β-actin (1:2000, #4970; CST). Then, the membranes were incubated with horseradish peroxidase-conjugated secondary antibodies (Shengxing Biological) before visualization using enhanced chemiluminescence (Millipore).

### Statistical Analysis

Statistical analysis was performed using GraphPad Prism 8.0. All data are presented as the mean ± standard deviation (SD) unless otherwise indicated. Data normality was examined by Shapiro-Wilk test (n < 10). For normally distributed data, comparisons between 2 groups were performed using Student’s t-test, and comparisons among multiple groups were performed using ANOVA followed by *post hoc* Bonferroni correction. Data homoscedasticity was examined by the Bartlett’s and Brown-Forsythe test. Non-homoscedastic data was analyzed by the Brown-Forsythe ANOVA test followed by Dunnett’s T3 multiple comparisons test. For non-normal data comparisons were performed by the Mann-Whitney U test or the Kruskal-Wallis test followed by Dunn’s multiple comparison test. P <.05 was considered statistically significant.

## Results

### Orthodontic Tooth Movement Induces Inflammation in Periodontal Tissues

**Figure 1:**
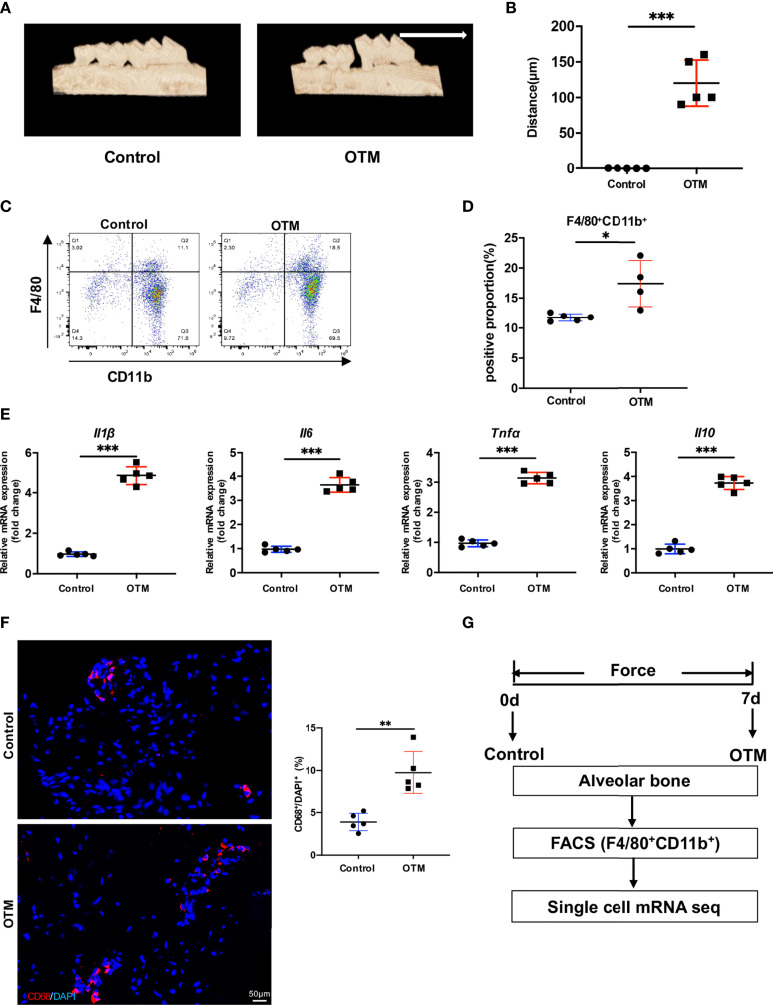
Orthodontic tooth movement induces inflammation in the periodontal tissues. **(A)** Representative 3D micro-CT reconstruction of murine OTM model. (White arrow: direction of force and tooth movement). **(B)** The distance of OTM and **(C)** the ratio of CD11b^+^F4/80^+^ macrophages at day 0 and 7. Values are mean ± SD. n = 5. ***P < 0.001. **(C, D)** Representative flow cytometric analysis of CD11b^+^F4/80^+^ macrophages at day 0 and 7. Values are mean ± SD. n = 4 or 5. *P < 0.05. **(E)** mRNA expression of inflammatory factors of macrophages in murine alveolar bone at day 0 and 7. Values are mean ± SD. n = 5. ***P < 0.001. **(F)** Representative immuno-fluorescence staining and quantification of CD68^+^ macrophages in human periodontal tissue. Scale bar = 50μm. Values are mean ± SD. n = 5. **P < 0.01. **(G)** The flow chart of experimental design.

To mimic human OTM, we first applied a mechanical device for force application in mice for 7 days. Micro-CT analysis showed that the distance between the first and second molars significantly increased after 7 days of tooth movement, which suggests successfully established murine OTM model (**Figures 1A, B**). Then, to investigate the participation of macrophages, we used FACS analysis to examine the proportion of F4/80^+^CD11b^+^ macrophages in the alveolar bone around the first molars on day 7. FACS analysis showed that macrophages significantly increased after 7 days of OTM (**Figures 1C, D**). Real-time RT-PCR analysis indicated that the mRNA expression levels of macrophage inflammatory factors, including *Tnfα*, *Il1β*, *Il6*, and *Il10*, increased after force application (**Figure 1E**). IF staining showed that the number of CD68^+^ macrophages augmented in human periodontal tissues after force application (**Figure 1F**). Together, these results suggested that macrophages accumulate and polarize during OTM, which may play an important role in orthodontic remodeling.

### Single-Cell Analyses Identify Macrophage Subsets in the Murine Alveolar Bone

To fully explore the heterogeneity of macrophages during OTM, we first performed scRNA-seq of F4/80^+^CD11b^+^ macrophages isolated from the alveolar bone tissues of mice in the control group on the 10x Genomics platform (**Figure 1G**). After applying quality control filters, 3 903 cells from the control group were used for subsequent analysis (**Figures S1A, B**).

**Figure 2:**
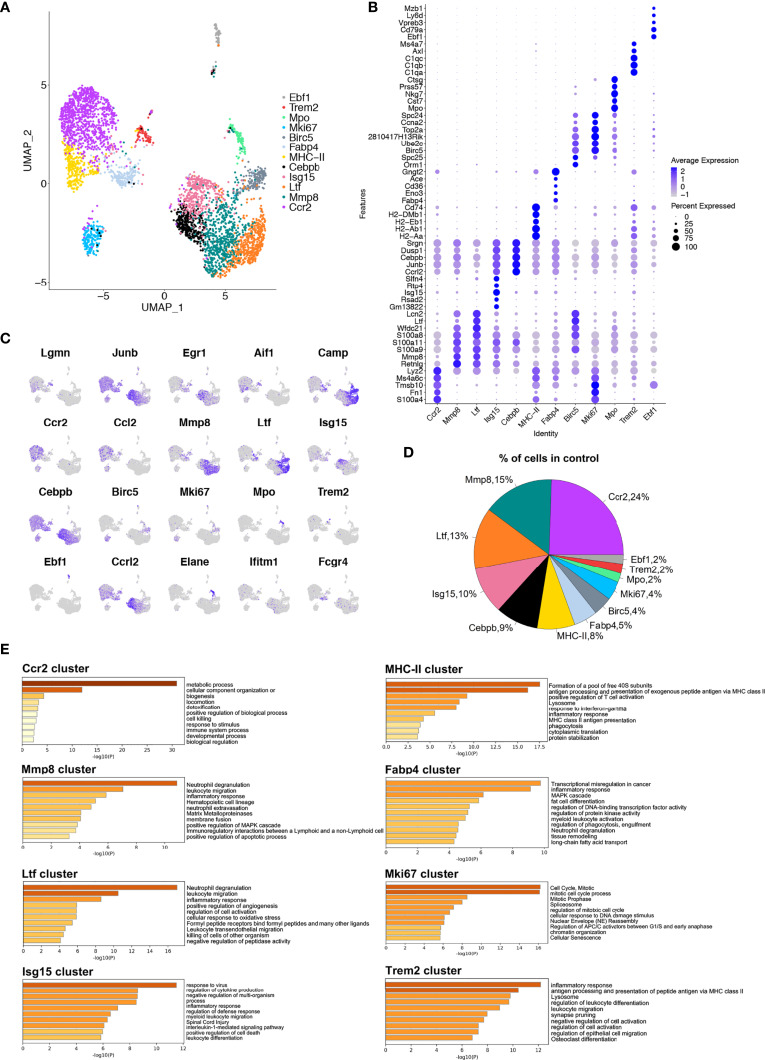
Single-cell analyses show macrophage subsets in murine alveolar bone. **(A)** UMAP plot of cells in control group. 12 (0 to 11) clusters were identified. Dimensional reduction was performed with principal component analysis (PCA) and visualization using UMAP plots. **(B)** Dot plot of differentially expressed genes in each cluster. **(C)** Feature plots depicting single-cell gene expression of individual genes. **(D)** Proportions of 12 different macrophage clusters. **(E)** The top 60-65 markers (adj. p-value < 0.05) per cluster were used to identify the functional enrichment categories using Metascape. Pathway enrichment is expressed as the -log10(P) adjusted for multiple comparison.

In the control group, uniform manifold approximation and projection (UMAP) dimensionality reduction analysis identified 12 clusters (**Figure 2A**). The top 25 differentially expressed genes (DEGs) for each cluster are presented (**Figure S2A**). To further understand the unique transcriptome features of each cluster, enrichment of the functions and signaling pathways of genes in each cluster was analyzed by Metascape (17) (**Figure 2E** and **Figure S3A**). Cluster 0 (termed the *Ccr2* cluster) had high expression levels of *Ccr2, Ccl2, S100a4*, and *Fn1*. Its biological function was related to ‘metabolic process’, ‘cell killing’, and ‘response to stimulus’. Cluster 1 (termed the *Mmp8* cluster) had high expression levels of *Retnlg, Mmp8* and *Mmp9*. Its enriched function was related to ‘neutrophil degranulation’, ‘leukocyte migration’, and ‘inflammatory response’. Matrix metalloproteinases (MMPs) are the main extracellular matrix (ECM) enzymes responsible for the degradation of collagen and other proteins relating to their ability to regulate cytokines. Cluster 2 (termed the *Ltf* cluster) had high expression levels of the *Ltf, Camp, Spp1*, and *Lcn2* genes. The main functions of this cluster included ‘neutrophil degranulation’, ‘leukocyte migration’, and inflammatory response’. Cluster 3 (termed the *Isg15* cluster) had high *Isg15, Slfn4, Slfn1*, and *Ifit3* genes. Its biological function was related to ‘regulation of defense response’, ‘defense response to virus’, and ‘response to interferon-beta’. Cluster 4 (the *Cebpb* cluster) had high expression levels of the *Il1b, Csf3r, Egr1, Ccrl2*, and *Cebpb* genes. Its enriched pathways were related to ‘inflammatory response’, ‘myeloid leukocyte migration’, and ‘response to IL-1’. Cluster 5 (termed the *MHC-II* cluster) had higher expression of *H2-Aa, Cd74, H2-Ab1, H2-Eb1*, and *H2-DMb1*. It is involved in ‘antigen processing and presentation’ and ‘inflammatory response’. Cluster 6 (termed the *Fabp4* cluster) had high expression of *Fabp4*, *Apoe*, *Fcgr4*, and *Ace.* Its enriched pathways were related to ‘fat cell differentiation’, ‘the inflammatory response’ and ‘tissue remodeling’, suggesting that this is the main cluster that ties the immune response to the regulation of lipid metabolism. Cluster 7 (termed the *Birc5* cluster) had high *Ube2c, Stmn1, Birc5, Ltf*, and *Serpinb1a* expression. The biological function of this cluster was related to the ‘cell cycle (mitosis)’ and ‘DNA packaging’. In cluster 8 (termed the *Mki67* cluster), the highly expressed genes were similar to those in cluster 7, including *Stmn1, Ube2c, Birc5, Tubb5*, and *Top2a*. The function of Cluster 8 was similar to that of Cluster 7, including ‘cell cycle’ and ‘response to DNA damage stimulus’. Cluster 9 (termed the *Mpo* cluster) had high expression levels of genes such as *Elane, Mpo, Prtn3, Ctsg, and Nkg7.* The biological function of this cluster was nucleoside triphosphate metabolism and interleukin-8 production. Cluster 10 (termed the *Trem2* cluster) had high expression of *C1qa, Trem2, Pf4* and *Vcam-1*. The distinctive pathway of ‘osteoclast differentiation’ was noticed in this cluster. Previous research has shown that triggering receptors expressed on myeloid cells 2 (*Trem2*) regulates osteoclast differentiation with impaired bone resorption capability in the periodontitis environment (18). We considered this cluster to be bone-resident macrophages with osteoclastogenic potential. In contrast, cluster 11 (termed the *Ebf1* cluster) was related to B cell proliferation and lymphocyte differentiation, with high expression of genes such as *Ebf1, Cd79a*, and *Cd79b* (**Figures 2B, C, E**). We then detected the proportion of each cluster in the steady-state murine alveolar bone tissue and found that the major cluster was the *Ccr2* cluster (**Figures 2D**). These data suggest the existence of distinctive macrophage clusters with specific functions in murine alveolar bone in the steady-state (no force application or OTM).

### CellChat and Single-Cell Trajectories Reveal Cell Communication and Developmental Relationships Among Macrophage Clusters in the Steady-State

**Figure 3:**
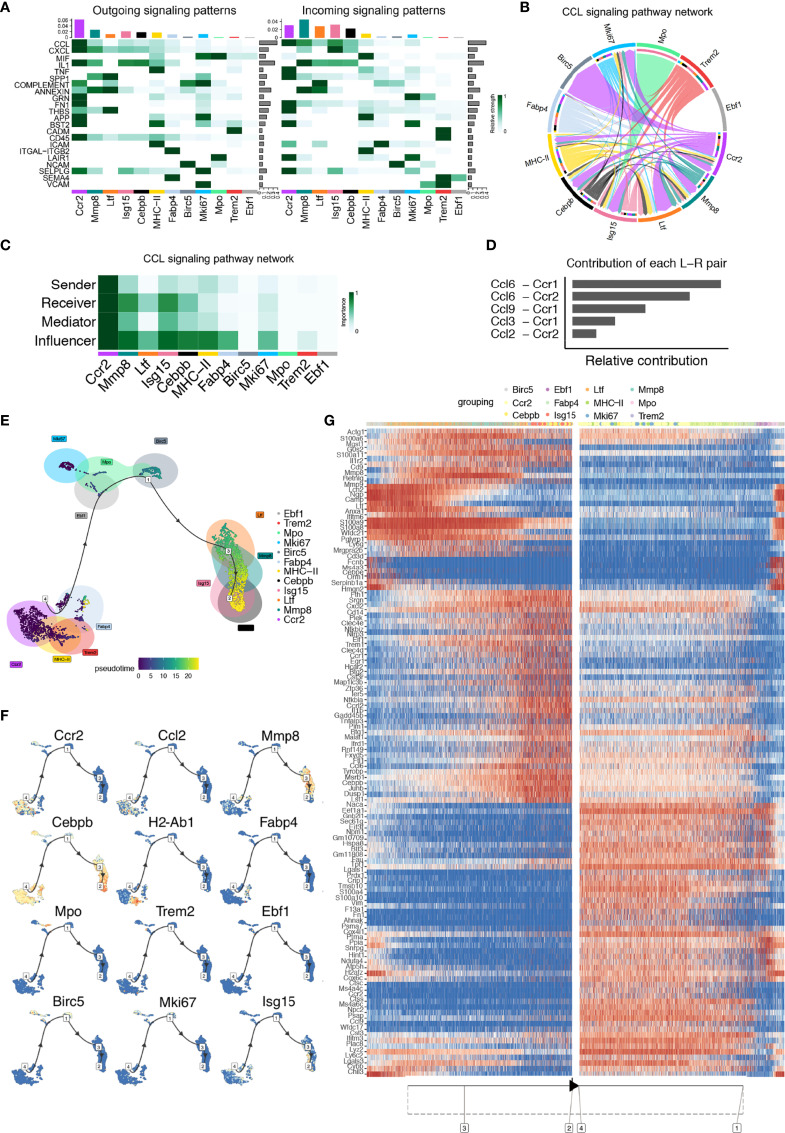
CellChat and Single-cell trajectories reveal cell communications and developmental relationships among macrophage clusters in the steady-state. **(A)** Heatmap shows the relative strength of each signal pathway network for each cluster with both incoming and outgoing signaling patterns. **(B)** Inferred CCL Signaling pathway network of steady-state clusters. **(C)** Heatmap of the CCL Signaling network displaying relative importance of each cell group ranked according to the computed four network centrality measures. **(D)** Relative contribution of each ligand-receptor pair as it affects the overall communication network of the CCL signaling pathway. **(E)** Differentially expressed genes between clusters were used to generate hypothetical developmental relationships using Dynverse. **(F)** Representative gene expression plotted as a function of pseudotime. **(G)** Heatmap of differentially expressed genes ordered based on their simple kinetics through pseudotime using Dynverse.

To explore the interactions between cells among the steady-state macrophage clusters, we applied CellChat to infer and analyze intercellular communication networks (15). CellChat detected 22 significant pathways between 12 macrophage clusters in steady-state alveolar bone tissue, among which the CCL signaling pathway exhibited the most prominent outgoing and incoming signaling patterns (**Figure 3A**). In-depth exploration of the CCL signaling pathway indicated that the *Ccr2* cluster exhibited high expression of the major sender, receiver, mediator, and influencer of the CCL signaling pathway (**Figures 3B, C**). Notably, the contribution of each ligand-receptor showed that the most significant L-R pairs were CCL6-CCR1 and CCL6-CCR2 (**Figure 3D**).

Seurat-defined clusters were superimposed on a pseudotime trajectory produced by the Dynverse algorithm to examine single-cell gene trajectories to determine the developmental relationships among these macrophage clusters. Three differentiation stages were identified. The *MHC-II* cluster was set as the origin cluster due to its high expression levels of antigen-presenting genes such as *H2-Aa, H2-Ab1, and H2-Eb1*, prevalent in normal macrophages. Trajectory analysis found that the *Ccr2, MHC-II, Trem2* and *Fabp4* clusters occupied the initial developmental stage (Phase 1), including 44.6% of total macrophages. The *Mki67, Mpo, Birc5* and *Ebf1* clusters, shown as the second development stage (Phase 2) could arise from Phase 1 and represented 11.3% of total macrophages. The *Ltf, Mmp8, Isg15* and *Cebpb* clusters, crucial for the inflammatory response and defense response, were located in the final development stage (Phase 3), possibly arising from Phase 2, presenting 44.1% of the total macrophages (**Figure 3E**).

Gene expression levels were plotted as a function of pseudotime by Dynverse to track changes across different macrophage states (**Figure 3F**). Genes such as *Ccr2, Ccl2, Lgmn* and *H2-Ab1* were highly expressed in Phase 1, and their enriched function was related to ‘cellular response to stress’ and ‘inflammatory response’. Since CCR2, a chemokine receptor, is critical for migration and expressed by circulating monocytes, the enrichment of this gene suggested that these clusters may be monocyte-derived. The expression of genes such as *Mki67, Birc5*, and *Mpo* was low in Phase 1. Still, it increased in Phase 2 with relevant functions in ‘cell cycle’ and ‘chromatin organization’, illustrating that clusters in Phase 2 were a proliferative macrophage population. Genes such as *Ccl6, Cxcl2, and Il-b* were highly expressed in Phase 3, with pathways related to ‘inflammatory response’, ‘neutrophil degranulation’, and ‘cytokine production regulation’, demonstrating their function in regulating the immune response (**Figure 3G**). These gene expression changes across macrophage clusters could define and mark different macrophage subsets and states according to their expression patterns.

### Single-Cell Analyses Identify Macrophage Subsets in Murine Alveolar Bone After Orthodontic Force Application

**Figure 4:**
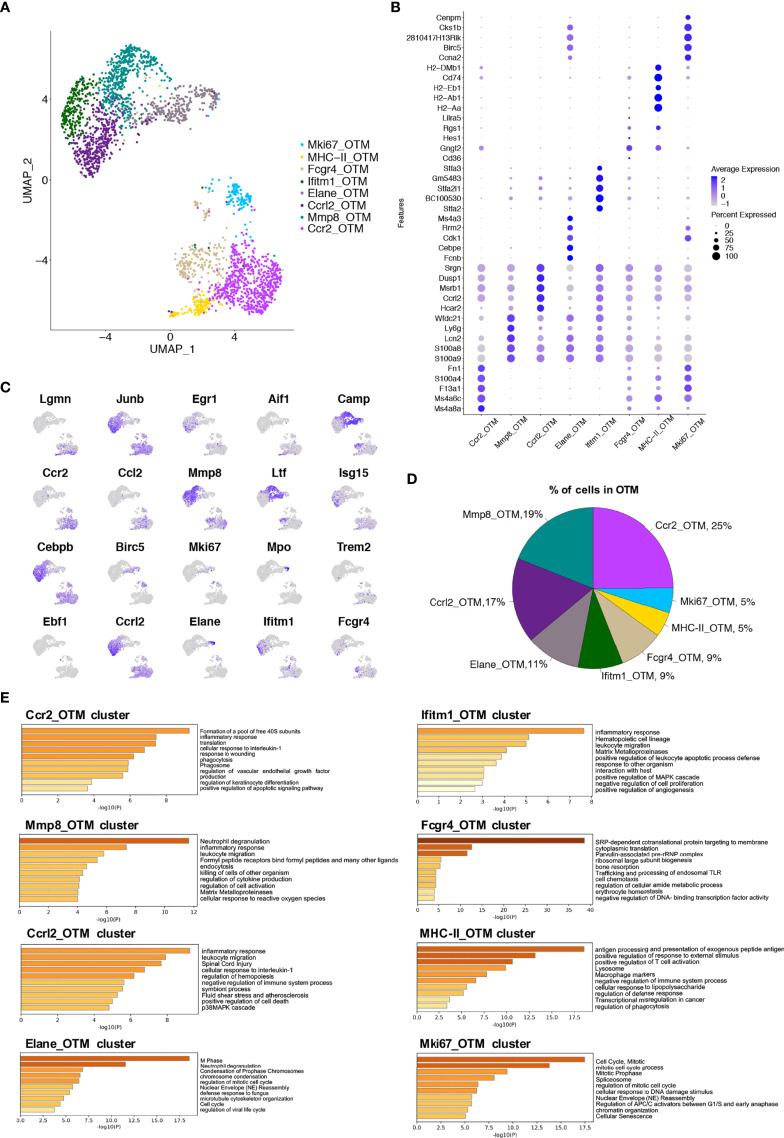
Single-cell analyses show macrophage subsets in murine alveolar bone after orthodontic force application. **(A)** UMAP plot of cells in OTM group. 8 (0 to 7) clusters were identified. Dimensional reduction was performed with principal component analysis (PCA) and visualization using UMAP plots. **(B)** Dot plot of conserved differentially expressed genes in each cluster. **(C)** Feature plots depicting single-cell gene expression of individual genes. **(D)** Proportions of 8 different macrophage clusters. **(E)** The top 60-65 markers (adj. p-value < 0.05) per cluster were used to identify the functional enrichment categories using Metascape. Pathway enrichment is expressed as the -log10(P-adjusted) for multiple comparison.

After establishing macrophage clusters in the steady-state, we compared their dynamic changes in macrophages during OTM. Macrophages were isolated from murine alveolar bone tissue after 7 days of the force application. After applying quality control filters, 2 534 cells from the OTM group were used for subsequent analysis (**Figures S1C, D**). The UMAP analysis revealed 8 clusters in this group (**Figure 4A**). The top 25 differentially expressed genes for each cluster are shown (**Figure S2B**). Cluster 0 (termed the *Ccr2_OTM* cluster) showed high expression of *Ccr2, Ccl9* and *Fn1*. As in the steady-state, we attributed this cluster to monocyte-derived macrophages. Notably, the pathway enriched in this cluster, ‘cell response to wounding’ and ‘cellular response to interleukin-1’, might be induced by orthodontic force mediated macrophage recruitment. Cluster 1 (termed the *Mmp8_OTM* cluster) had a relatively high expression of genes, including *Mmp9, Mmp8, Ly6g, Ltf*, and *Spp1*. The enrichment analysis identified functions and pathways such as ‘inflammatory response’ and ‘leukocyte migration’. Cluster 2 (termed the *Ccrl2_OTM* cluster) had high expression of genes such as *Ccrl2, Cxcr2, Cs43r*, and *Egr1*, with enrichment of functions related to the inflammatory response, including ‘cytoplasmic translation’ and ‘bone resorption’. Cluster 3 (termed the *Elane_OTM* cluster) had a high expression of genes necessary for cell division, such as *Hmgn2, Ube2c, Tubb5*, *Ltf* and *Camp.* Its enriched pathways were related to cell proliferation, such as ‘cell cycle’, ‘DNA replication’, and ‘nucleotide metabolic process’. This cluster, therefore, may assume functions in proliferation and self-renewal. Cluster 4 (termed the *Ifitm1_OTM* cluster) highly expressed the stefin genes (*Stfa2, BC100530*, and *Gm5483)* as well as *Asprv1, Ifitm1*, and *Mmp9.* Its biological function is related to classic inflammatory pathways, including ‘inflammatory response’ and ‘leukocyte migration’. Cluster 5 (termed the *Fcgr4_OTM* cluster) had high expression of genes such as *Cd83, Rgs1, Fcgr4* and *Apoe*. This cluster showed increased function in ‘response to external stimulus’ and ‘antigen processing and presentation’. Cluster 6 (termed the *MHC-II_OTM* cluster) showed relatively high expression of genes such as *Cd74, H2-Aa, H2-Ab1*, and *H2-Eb1*. The biological functions mainly included ‘antigen processing and presentation’. Cluster 7 (termed the *Mki67_OTM* cluster) had high expression of the genes *Lgals1, Tmsb10, Tubb5*, and *Tubb1b.* The upregulated biological functions were related to proliferation-related and translational-ribosomal pathways (**Figures 4B, C, E**). The proportions of each cluster in the OTM murine alveolar bone tissue were calculated, and the major cluster was still the *Ccr2* cluster (**Figure 4D**). These data revealed the existence of cluster-specific functions in macrophages after force application.

### CellChat and Single-Cell Trajectories Reveal Cell Communication and Developmental Relationships of Macrophages After Orthodontic Force Application

**Figure 5:**
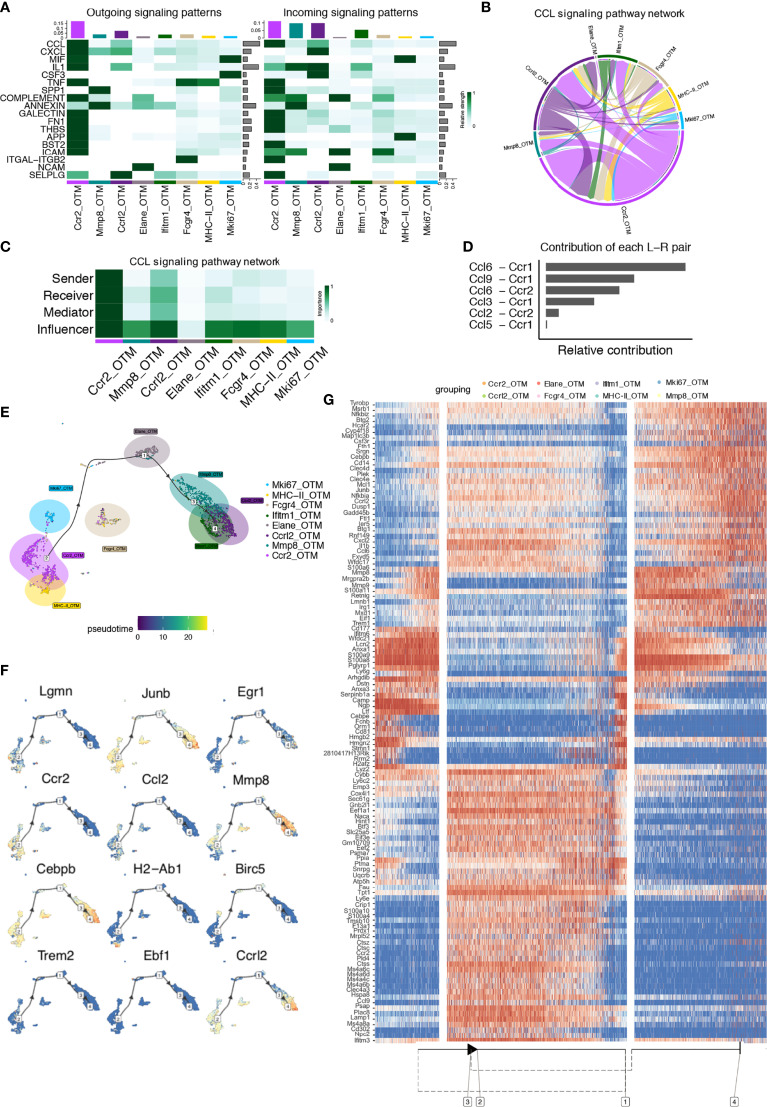
CellChat and Single-cell trajectories reveal cell communications and developmental relationships of macrophages after orthodontic force application. **(A)** Heatmap shows the relative strength of each signal pathway network for each cluster with both incoming and outgoing signaling patterns. **(B)** Inferred CCL Signaling pathway network of case clusters. **(C)** Heatmap of the CCL Signaling network displaying relative importance of each cell group ranked according to the computed four network centrality measures. **(D)** Relative contribution of each ligand-receptor pair as it affects the overall communication network of the CCL signaling pathway. **(E)** Differentially expressed genes between clusters were used to generate hypothetical developmental relationships using Dynverse. **(F)** Representative gene expression plotted as a function of pseudotime. **(G)** Heatmap of differentially expressed genes ordered based on their simple kinetics through pseudotime using Dynverse.

CellChat analysis of the OTM clusters was performed. A total of 18 significant pathways were detected, among which the CCL signaling pathway had the highest relative strength index in both incoming and outgoing signaling patterns (**Figure 5A**), similar to the steady-state clusters. Likewise, the CCL signaling pathway was most significantly upregulated in the *Ccr2_OTM* cluster, playing major roles as a sender, receiver, mediator, and influencer (**Figures 5B, C**). Ligand-receptor analysis showed that the CCL6-CCR1 and CCL9-CCR1 pairs made the most significant relative contributions (**Figure 5D**). *Ccr1* is mainly expressed in monocytes, T cells, dendritic cells, and neutrophils, whereas *Ccr2* is mainly expressed in macrophages in the immune system (19, 20). Previous research has demonstrated that *Ccr2*, instead of *Ccr1*, is the sole receptor responsible for monocyte mobilization from the bone marrow and recruitment to inflamed sites (21).

Trajectory analysis was conducted on these clusters. Similar to the steady-state, the *Ccr2_OTM* and *MHC-II_OTM* clusters also occupied at the original point of the branch (Phase 1), with 42% of total cells residing in this phase, a 2.6% decrease. The *Elane_OTM* cluster accounting for 13.9% of total cells from Phase 2 might arise from Phase 1, rising 2.6% compared to the steady-state, and the *Mki67_OTM* cluster and *Fcgr4_OTM* cluster seemed to be in the transition phase between phases 1 and 2. In addition, the *Mmp8*, *Ccrl2* and *Ifitm1* clusters occupied the end of the developmental stage (Phase 3), which accounted for 44.1%, with no significant difference compared to the steady-state. This analysis demonstrated that the abundance of macrophages increased in the proliferative states and decreased in the primary state after orthodontic force application (**Figure 5E**).

Gene expression levels were plotted as a function of pseudotime in Dynverse to track changes across different macrophage states (**Figure 5F**). In contrast to steady-state clusters, the expression patterns of genes in the clusters after force application were different. *Ccr2, Il1b, Ctss, and Ccl9* expression peaked in Phase 1 and then dropped in Phase 2. Pathways that were enhanced in Phase 1 included ‘neutrophil degranulation’ and ‘cellular response to chemical stress’. The expression levels of the *Prtn3, Elane, and Ptma* genes were highest in Phase 2 and then decreased in the other phases. Its functions include the cell cycle and neutrophil degranulation. Genes such as *S100a8, Erg1, Mmp9*, and *Mmp8* were more evident in Phase 3. Its enriched functions were related to the inflammatory response and IL-8 production (**Figure 5G**).

### Macrophage Genes and Functions Switch After Force Application

**Figure 6:**
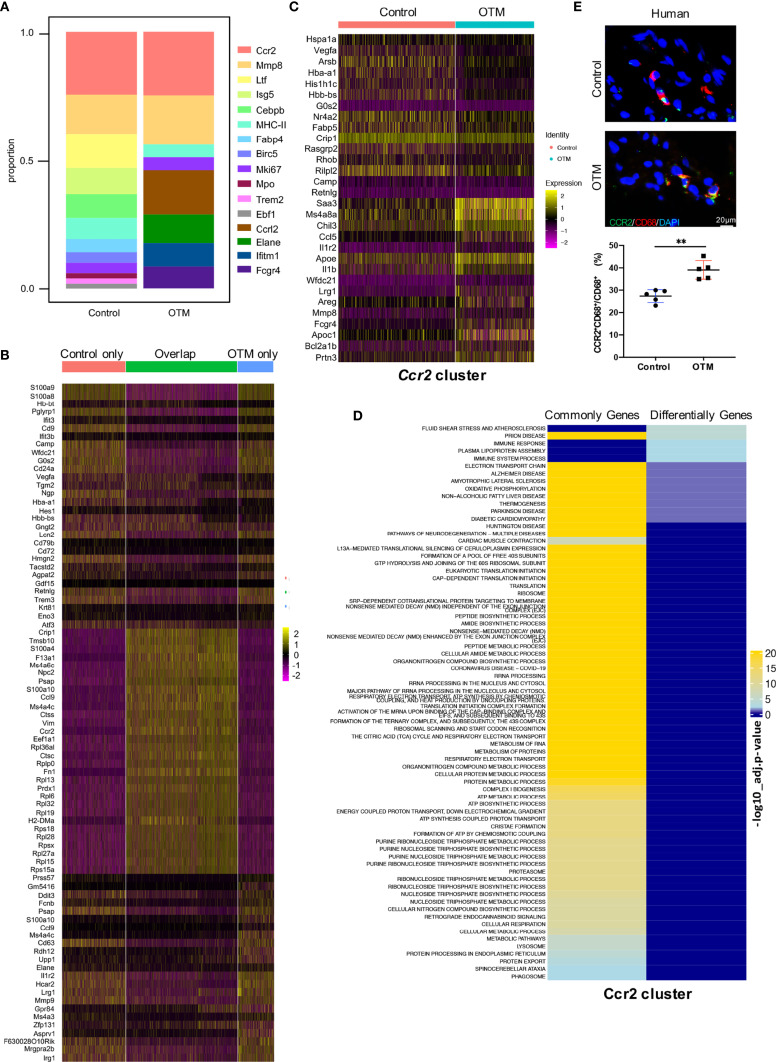
High-dimensional analyses reveal both unique and shared macrophage genes and functions after force application. **(A)** Bar chart of the relative proportions of clusters from two groups. **(B)** Heatmap of the top 30 differentially expressed genes in control only, OTM only, and overlapping populations. **(C)** Heatmap of the top 15 differentially expressed genes in *Ccr2* cluster between two groups. **(D)** Pathway analysis (gProfiler) of commonly and differentially expressed genes in *Ccr2* cluster. **(E)** Representative immunofluorescence staining and quantification of CD68^+^CCR2^+^ macrophages in the human periodontal tissue. Scale bar = 20μm. Values are mean ± SD. n = 5. **P < 0.01.

The macrophage compositions under each condition were compared. After force application, 4 clusters emerged and 7 clusters disappeared. The *Ccr2* cluster constituted the largest subpopulation in either the control or OTM conditions (**Figure 6A**). We compared macrophages between the two groups and selected unique and shared genes for further analyses. The shared genes highly expressed in the two groups included *Crip1*, *Ctss*, *S100a4*, *Ccl9*, and *Fn1*. The enriched functions included ‘multiple metabolic processes’, ‘protein translation’, and ‘biosynthetic process’. Genes highly expressed only in the control group included *S100a8*, *S100a9*, *Itif3* and *Camp*, with related pathways of ‘IL-17 signaling’, ‘neutrophil degranulation’, and ‘producing antimicrobial peptides’. In contrast, the unique genes in the OTM group included *Ddit3*, *Il1r2*, *Mmp9*, *Stfa2*, and *Stfa3*, with the distinguishing feature of ‘allergy’ and ‘endopeptidase regulation’, which revealed the activation of macrophages after OTM (**Figure 6B** and **Figure S4A**).

Four common clusters were defined in both groups, *Ccr2, Mmp8*, *MHC-II* and *Mki67* and differentially expressed genes in the four clusters were identified. Genes in the *Ccr2* cluster, including *Ccl5, Il-1b* and *Mmp8*, were involved in the inflammation process, and their expression levels significantly increased after force application (**Figure 6C**). There were more genes associated with antigen presentation expressed in the *MHC-II* cluster after OTM. The *Mmp8* cluster in the OTM has high expression levels of *Ngp*, *Ltf* and *Camp*, which were the characteristic genes related to inflammation response. The osteoclast differentiation-related genes *Acp5* and *Cxcl1* were highly expressed in the *Mki67* cluster after OTM, suggesting a possible effect on regulating bone remodeling (**Figures S5A–C**). Function enrichment analysis of the commonly and differentially expressed genes before and after force application in the *four* clusters was performed. In the *Ccr2* cluster, the functions of differentially expressed genes were related to fluid shear stress and the immune response. In contrast, the functions of commonly expressed genes were focused on ribosomes, metabolic processes, and biosynthetic processes. In the *Mmp8* cluster, the differentially expressed genes were related to translation, the cell cycle, and protection against neuronal diseases. In contrast, commonly expressed genes were focused on neutrophil granulation, cytoskeletal organization, etc. The *MHC-II* cluster consisted of differentially expressed genes with enhanced functions related to the inflammatory response, stress response, and chemokine signaling. Moreover, the *Mki67* cluster had fewer differentially activated pathways, mainly grouped into oxygen transport and rheumatoid arthritis immunity (**Figure 6D** and **Figures S6A–C**). The above results further confirmed that the *Ccr2* cluster played a key role during OTM, consistent with the above suspicion. In human periodontal tissues, we conducted IF staining to detect the *Ccr2* cluster. IF staining also demonstrated that CCR2^+^CD68^+^ macrophages were increased after force application, highlighting its relevance and significance in humans (**Figure 6E**).

### The CCR2^+^ Macrophage Cluster Promotes OTM in a CCL2/CCR2/p65 Axis-Dependent Manner

**Figure 7:**
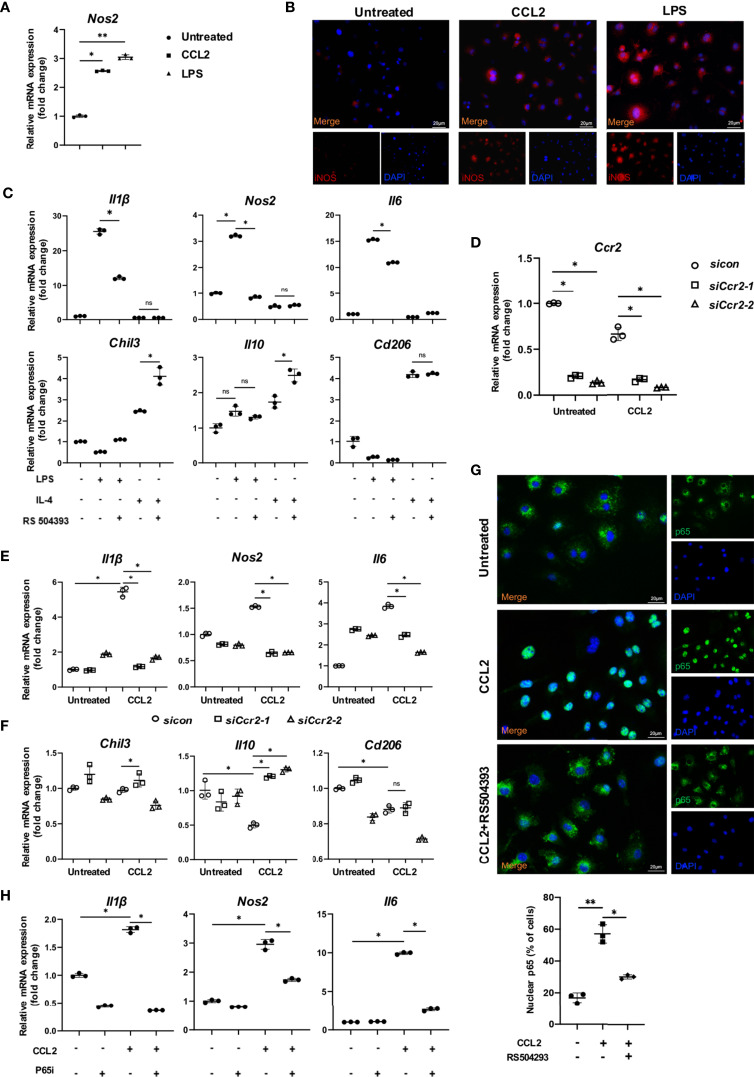
CCR2/CCL2 axis promoted pro-inflammatory macrophages through the phosphorylation of p65. **(A)** Relative mRNA expression of *Nos2* in untreated, CCL2 treated and LPS treated macrophages. Data is from 3 independent experiments. Values are mean ± SD. *P < 0.05. **P <0.01. **(B)** Representative immunofluorescence staining of iNOS in untreated, CCL2 treated and LPS treated macrophages. **(C)** Relative mRNA expression of inflammatory factors in macrophages treated with LPS or IL-4, and RS504393. Data is from 3 independent experiments. Values are mean ± SD. *P < 0.05. ns, no significance. **(D)** Relative mRNA expression of *Ccr2* in macrophages treated with CCL2 or not, and with *Ccr2* siRNA. Data is from 3 independent experiments. Values are mean ± SD. *P < 0.05. **(E, F)** Relative mRNA expression of pro-inflammatory **(E)** and anti-inflammatory factors **(F)** in macrophages treated with CCL2 or not, and with *Ccr2* siRNA. Data is from 3 independent experiments. Values are mean ± SD. *P < 0.05. ns, no significance. **(G)** Representative immunofluorescence staining of p65 in macrophages in untreated, treated with CCL2, and CCL2+RS504393. Data is from 3 independent experiments. Values are mean ± SD. *P < 0.05. **P <0.01. **(H)** Relative mRNA expression of inflammatory factors *Il6*, *Il1β* and *nos2* treated with CCL2 or p65 inhibitor. Data is from 3 independent experiments. Values are mean ± SD. *P < 0.05, ns, no significance.

To further explore the underlying mechanism of the CCR2^+^ macrophage cluster during OTM, we stimulated macrophages with CCL2 or LPS. Expression of *Nos2* mRNA increased after CCL2 or LPS treatment, which confirmed a pro-inflammatory role of the CCR2/CCL2 axis in macrophages (**Figure 7A**). IF staining confirmed the significant upregulation of iNOS in macrophages treated with CCL2 or LPS (**Figure 7B**). Further, the administration of RS504393, a selective CCR2 antagonist (22), demonstrated that blocking CCR2 could inhibit the pro-inflammatory effects induced by LPS. The mRNA analysis further showed a decrease of pro-inflammatory cytokines (*Il1β*, *Nos2*, and *Il6*) and increased reparative cytokines (*Il10* and *Cd206*) after incubation of RS504393 (**Figure 7C**). Next, we used *Ccr2*-siRNA to downregulate the expression level of *Ccr2* in macrophages. The expression levels of the pro-inflammatory macrophage markers *Il1β*, *Nos2*, and *Il6* were increased by CCL2 treatment and then decreased due to the silencing of *Ccr2* (**Figures 7D, E**). In contrast, the expression levels of the anti-inflammatory macrophage markers *Il10*, *Cd206* and *Chil3* were not significantly different (**Figure 7F**). Collectively, these findings suggest that CCR2 is involved in macrophage inflammatory functions, promoting the expression of pro-inflammatory cytokines.

**Figure 8:**
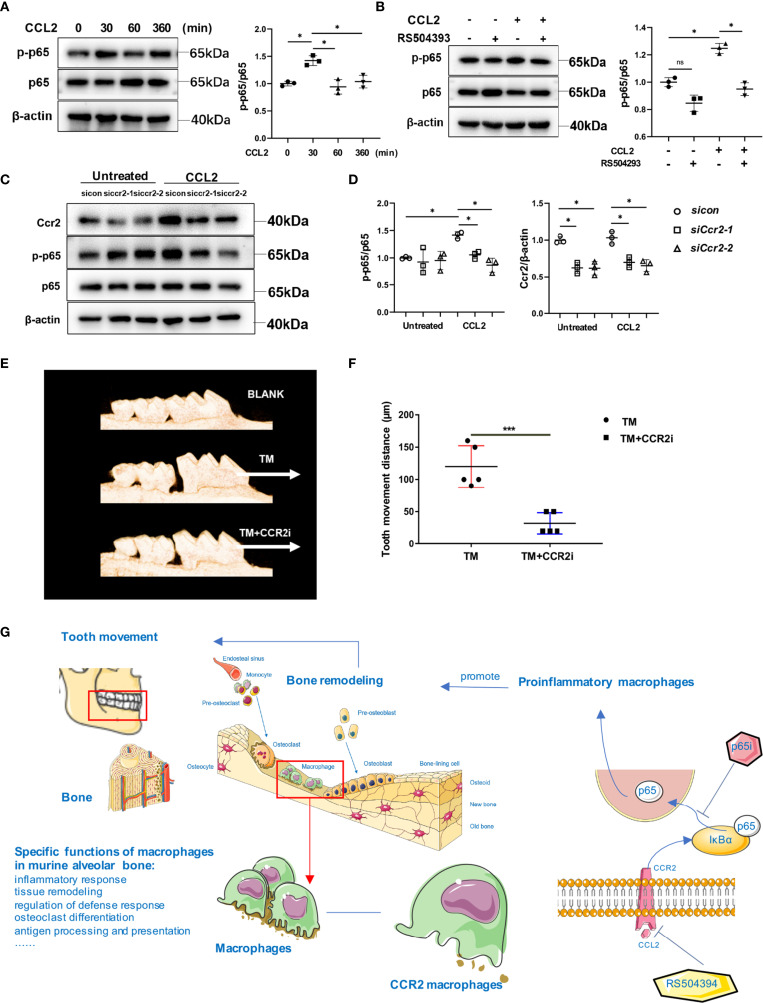
CCL2 treatment promoted the phosphorylation of p65 in macrophages. **(A)** Western blot analysis of p-p65 and p65 expression in macrophages treated with CCL2 for 0, 30, 60 and 360 minutes. Data is from 3 independent experiments. Densitometric quantitation with Image J. Values are mean ± SD. *P < 0.05. **(B)** Western blot analysis of p-p65 and p65 expression in macrophages treated with CCL2 and RS504393. Data is from 3 independent experiments. Densitometric quantitation with Image J. Values are mean ± SD. *P < 0.05. ns, no significance. **(C, D)** Western bolt analysis of p-p65 and p65 expression in macrophages treated with CCL2 or not, and with *Ccr2* siRNA. Data is from 3 independent experiments. Densitometric quantitation with Image J. Values are mean ± SD. *P < 0.05. **(E, F)** The distance of OTM in TM and TM+CCR2i group and representative 3D micro-CT reconstruction. White arrow: direction of force and tooth movement. Values are mean ± SD. n = 5. ***P < 0.001. **(G)** Graphic abstract of this study.

Next, to explore the mechanisms by which CCR2 promotes pro-inflammatory polarization, GSEA was performed in the *Ccr2* cluster between the control and OTM groups. The *Ccr2* cluster revealed enhanced effects of the cytokine-cytokine receptor interaction and chemokine signaling pathway after OTM, consistent with our previous results (**Figures S7A, B**). Moreover, the NF-κB pathway and inflammatory response were the top two pathways activated during OTM (**Figures S7C, D**). The transcription factor NF-κB plays central role in immune and inflammatory responses, bone development, and disease. The most common NF-κB prototype is the heterodimer of p65 and p50, with p65 as the major subunit (23). To delineate the role of CCR2 in modulating the NF-κB pathway in macrophages, we examined p65 expression in macrophages treated with CCL2 or RS504393. CCL2 significantly increased p65 accumulation in the nucleus, whereas RS504393 significantly reduced this activation (**Figure 7G**). Relative mRNA expression of the pro-inflammatory macrophage markers *Il1β*, *Nos2*, and *Il6* were upregulated by CCL2 treatment. This upregulation was reversed by p65 inhibitor treatment together with CCL2 (**Figure 7H**). Western blot analysis also proved that CCR2 activation could induce p65 phosphorylation in macrophages (**Figure 8A**), and CCR2 blockade with the inhibitor RS504393 could reduce the activation of p65 (**Figure 8B**). Moreover, CCR2 knockdown leads to a significant reduction of p65 phosphorylation without expression change of p65 under the stimulation of CCL2, which is consistent with the results above (**Figures 8C, D**). These findings indicate a CCR2 mediated macrophages pro-inflammatory function transition through phosphorylation of p65.

Subsequently, we confirmed the central role of the *Ccr2* cluster by continuously injecting peritoneally RS504393 into mice during OTM. RS504393 was injected into OTM mice based on a previous study, and no significant side effects were found during OTM in our study (11). There was a significant decrease in OTM distance after CCR2 inhibitor administration (**Figures 8E, F**). These results suggest that the *Ccr2* cluster is a virtual macrophage cluster for mechanical force-induced tooth movement and bone remodeling.

## Discussion

In this study, we used scRNA-seq combined with advanced bioinformatics analysis to evaluate macrophage heterogeneity under steady-state conditions and during OTM in alveolar bone tissues. The results indicate that macrophages in the murine alveolar bone can be categorized into different clusters with specific functions. We defined bone tissue-resident macrophages by functional analysis and gene expression characterization of all the clusters. CellChat analysis revealed that the CCL signaling pathway exhibited the most prominent signaling patterns in the *Ccr2* cluster. Comparison of the gene expression profiles and functions of *Ccr2* clusters before and after OTM revealed a significant increase of expression of inflammatory markers after OTM. Furthermore, we showed that the CCR2/CCL2 axis plays a crucial role in CCR2^+^ macrophages. Depletion of CCR2 led to OTM inhibition. Blocking CCR2 with a specific inhibitor or silencing CCR2 led to a decrease in pro-inflammatory factor expression, which was mediated by phosphorylation of p65 and activation of NF-κB (**Figure 8G**). By exploring the immunological mechanisms involved in tooth movement, individual treatment based on physiological characteristics may be applied to adjust and control the movement during orthodontic treatment; immunoreaction-induced pain during treatment may be alleviated by targeting specific pathways or mediators.

Both innate immunity and adaptive immunity coordinate govern bone absorption and reconstruction (24). However, excessive inflammation is implicated in bone injury and remodeling. Immunomodulatory approaches targeting specific cell types or factors may help the timely resolution of inflammation and modulate bone remodeling. Bone marrow macrophages are involved in removing apoptotic cells, particularly apoptotic osteoblasts, through a process named efferocytosis (25). This phagocytic process plays an essential role in bone tissue homeostasis under disease conditions and during new bone formation. Recent findings revealed that successful fracture healing is governed by carefully coordinated crosstalk between inflammatory and bone-forming cells, particularly macrophages and mesenchymal stem cells (MSCs) (26). Vi et al. verified that rejuvenation of bone repair in mice was orchestrated by macrophage cell secretion (27). Macrophage-derived paracrine signaling molecules such as oncostatin M, prostaglandin E2 (PGE2), and bone morphogenetic protein-2 (BMP2) have been proved to have critical roles in modulating bone remodeling (28, 29). Overall, although the essential roles of macrophages during bone formation and the healing process are observed, the mechanisms remain unclear. The conventionally defined distinct subsets, classically and alternatively activated macrophages, are insufficient to meet the demands of detailed mechanistic explorations. The recent emergence of single-cell technology provided a practical approach to elucidate previously hidden information among individual cells by isolating cells directly from human or animal tissues. By scRNA-seq analysis, we identified different clusters of macrophages with specific functions in murine alveolar bone for the first time. This approach enables in-depth investigation of macrophage involvement in bone tissue in steady-state conditions and after force applications. However, OTM is a long process with dynamic changes in macrophage in quantity and heterogeneity. While the current study focused on 7-day movement in mice, multiple time points of tooth movement should be examined in the future.

The prevailing dogma is that all macrophages originate from circulating monocytes. Recently, this theory has been challenged by lineage tracing studies, which proved that many tissue-resident macrophages arise from embryonic precursors (30). The collaboration between infiltrating and resident macrophages has been proven to contribute to optimal fracture healing (31). Resident macrophages in osteal tissues have been characterized in several studies with F4/80^+^Mac-2^−/low^ cells that reside within three cell diameters of a bone surface proved to be an integral component of bone tissue (31, 32). Simultaneously, they play a novel role in bone homeostasis by regulating osteoblast function. Nevertheless, the detailed features and functions of bone resident macrophages remain unclear. Here, we identified a cluster of macrophages with relatively high *Trem2, Pf4*, and *Vcam-1* genes expression as bone-resident macrophages. Triggering receptors expressed on myeloid cells 2 (*Trem2*) could regulate osteoclast differentiation *via* intercellular ROS signals during periodontitis (18). It is also considered a marker of resident macrophages in atherosclerosis. Platelet factor 4 (*Pf4*), which has been regarded as specific for megakaryocytes and platelets, is also highly expressed in the main population of vascular resident macrophages (33). Vascular cell adhesion protein-1 (*Vcam*-1), a factor that macrophages can express in steady-state, is essential for HSC retention. Macrophages present in erythroblasts islands were described as vital for HSC maintenance and considered BHSC niche macrophages (7). Therefore, it is reasonable to assume that this cluster is resident, whereas our identification of bone-resident macrophages may need further confirmation.

Here, we revealed the essential role of the *Ccr2* cluster in bone injury through scRNA-seq analysis. Both in the steady-state and OTM conditions, CCR2^+^ macrophages accounted for the largest proportion of all macrophages, with metabolic process functions, cell killing, and response to stimulus. More functions were activated after force application in this cluster, such as inflammatory response, cellular response to interleukin-1, response to wounding, etc. In fracture models, healing is delayed at injury sites due to the loss of CCR2-dependent monocyte recruitment (34). Furthermore, impaired callus formation was observed after treatment with CCR2 small molecule inhibitors in fracture models (35). In contrast, Batoon et al. considered that no difference in bone repair efficiency in CCR2-deficient mice compared to controls when assessing the healing of stabilized tibial injury, and the density of F4/80^+^ macrophages and the quantity of regenerating bone tissue at the injury site are not compromised by CCR2 deficiency (36). We investigated the role of CCR2 in the bone remodeling process during OTM with the pharmacological blockade of CCR2 signaling, RS504393, a CCR2 antagonist that has been shown to specifically inhibit CCL2/CCR2 interactions, which significantly decreased the OTM distance at 7 days. Our result suggested the necessity of monocyte-derived macrophages in the bone remodeling process during OTM. Repeated administration of RS504393 reduced the mRNA and/or protein levels of factors such as IL-1β, IL-18, IL-6, and iNOS and enhanced opioid analgesic potency (37). Moreover, no significant toxic effects were found in mice during the administration of RS-504393. Recruited macrophages are not required or at least are not recruited *via* a CCR2-dependent mechanism in some bone injury models. However, in OTM, monocyte-derived macrophages are of great importance, dominating both quantity and function (23). By exploring the heterogeneity of macrophages in OTM, the roles played by individual macrophage clusters might give insights into manipulating OTM rate through modulating immune function.

In conclusion, in this study we identified macrophage characteristics and biological functions at the transcriptome level during OTM through scRNA-seq. We revealed that CCR2^+^ macrophage cluster could regulate local microenvironment in both mice and humans. Our results indicated that the CCR2^+^ macrophage cluster might serve as a potential therapeutic target to accelerate OTM and shorten the treatment period. These findings provide a valuable means of understanding molecular mechanisms during OTM and may contribute to the development of the orthodontic treatment.

## Data Availability Statement

The datasets presented in this study can be found in online repositories. The names of the repository/repositories and accession number(s) can be found below: https://www.ncbi.nlm.nih.gov/geo/, GSE186185.

## Ethics Statement

The studies involving human participants were reviewed and approved by the Research Committee of the Affiliated Stomatological Hospital of Nanjing Medical University. Written informed consent to participate in this study was provided by the participants’ legal guardian/next of kin. The animal study was reviewed and approved by the Committee of Nanjing Medical University for Animal Resources. Written informed consent was obtained from the individual(s) for the publication of any potentially identifiable images or data included in this article.

## Author Contributions

HX and SZ designed the study and performed the experiments. Data collection and manuscript writing were done by HX. AS, HX, ZJ, and JG carried out statistical analysis and interpretation of the results. CX, HZ, and BY conceived and supervised the whole project. All authors read and approved the final manuscript.

## Funding

This study was supported by the National Natural Science Foundation of China (82071143 and 81571005) and the Key Medical Research Projects of Jiangsu Health Commission (ZDA2020003). We thank to the Priority Academic Program Development of Jiangsu Higher Education Institutions (PAPD, 2018‐87) and the Postgraduate Research & Practice Innovation Program of Jiangsu Province (KYCX20_1439).

## Conflict of Interest

The authors declare that the research was conducted in the absence of any commercial or financial relationships that could be construed as a potential conflict of interest.

## Publisher’s Note

All claims expressed in this article are solely those of the authors and do not necessarily represent those of their affiliated organizations, or those of the publisher, the editors and the reviewers. Any product that may be evaluated in this article, or claim that may be made by its manufacturer, is not guaranteed or endorsed by the publisher.
