# Supplementary material for: CCR2+ Macrophages Promote Orthodontic Tooth Movement and Alveolar Bone Remodeling
Source: Front Immunol. 2022 Feb 4;13:835986. doi: 10.3389/fimmu.2022.835986 (PMC8854866; doi:10.3389/fimmu.2022.835986)
Supplement: Supplementary file 1 [file Image_1.pdf]

Supplementary Figure 1

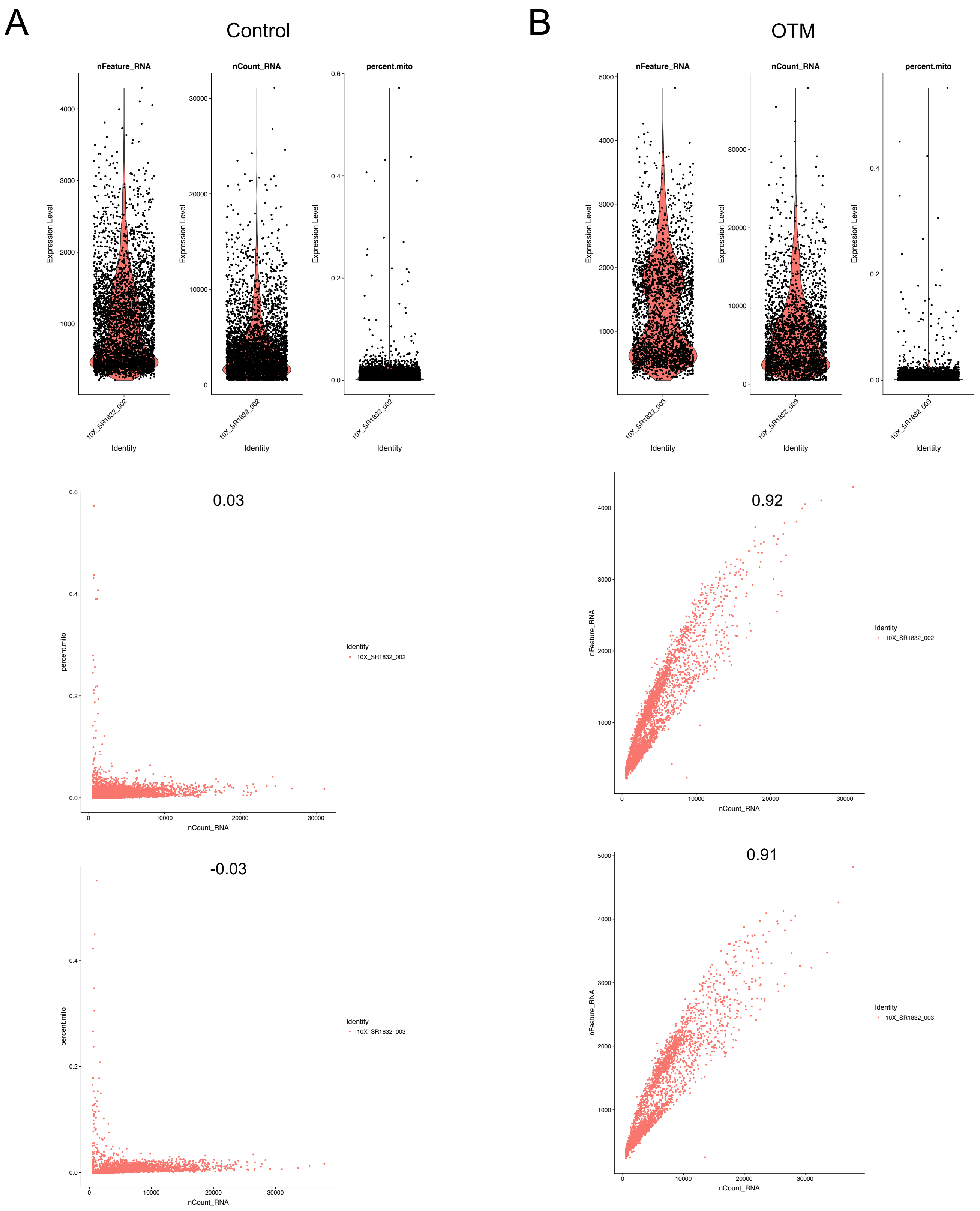

Supplementary Fig. 1 Quality control of the alveolar bone tissue scRNA-seq data.
(A, C) The violin plots showed the distribution of feature RNA, count RNA and mitochondrial RNA proportions in control and OTM group.
(B, D) FeatureScatter plot exhibited the relationship between Feature RNA and count RNA, as well as mitochondrial RNA and count RNA in two groups.

Supplementary Figure 2

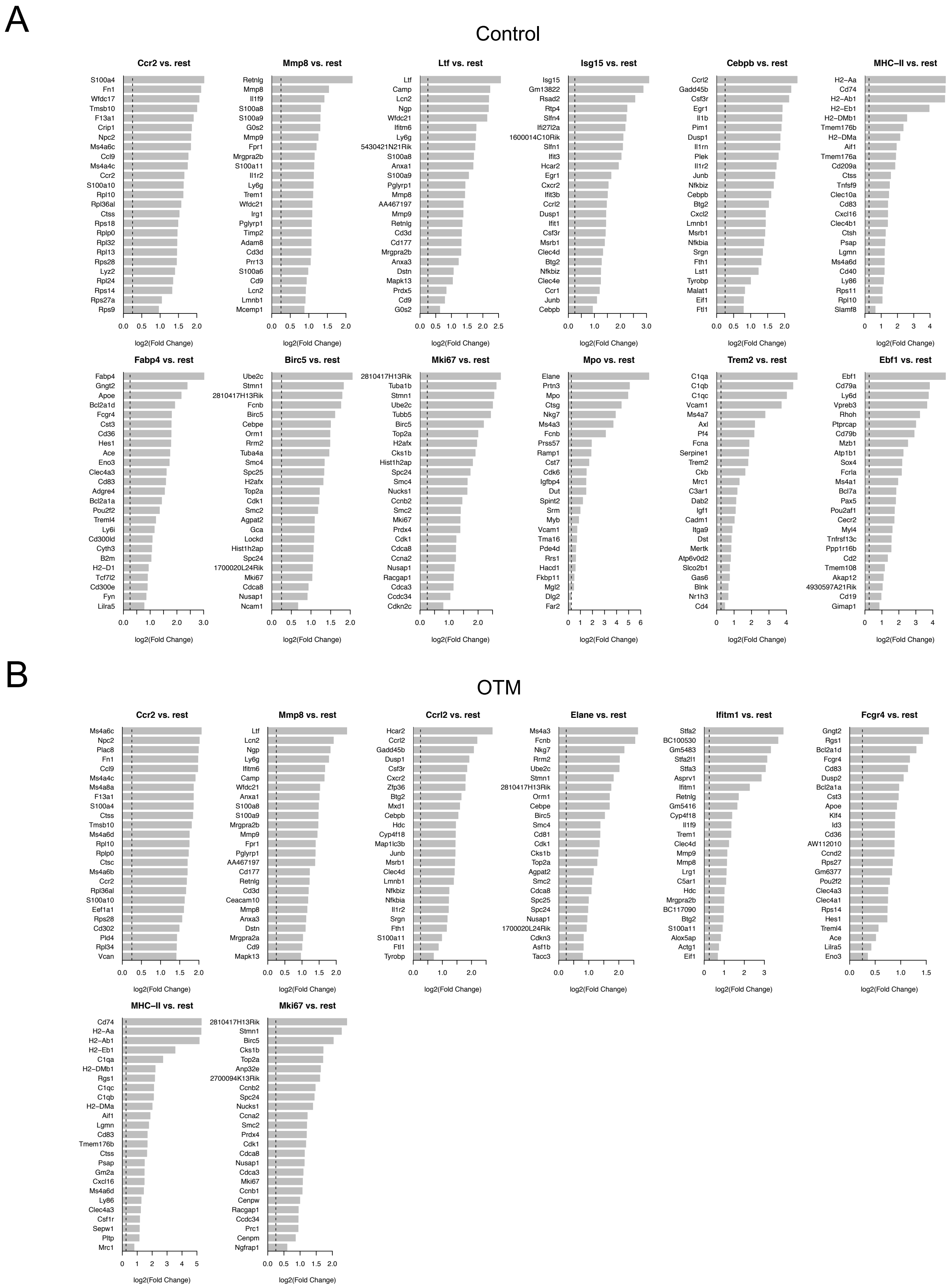

Supplementary Fig. 2 Barplot of top 25 differential expressed genes in control and OTM group.

Supplementary Figure 3

A

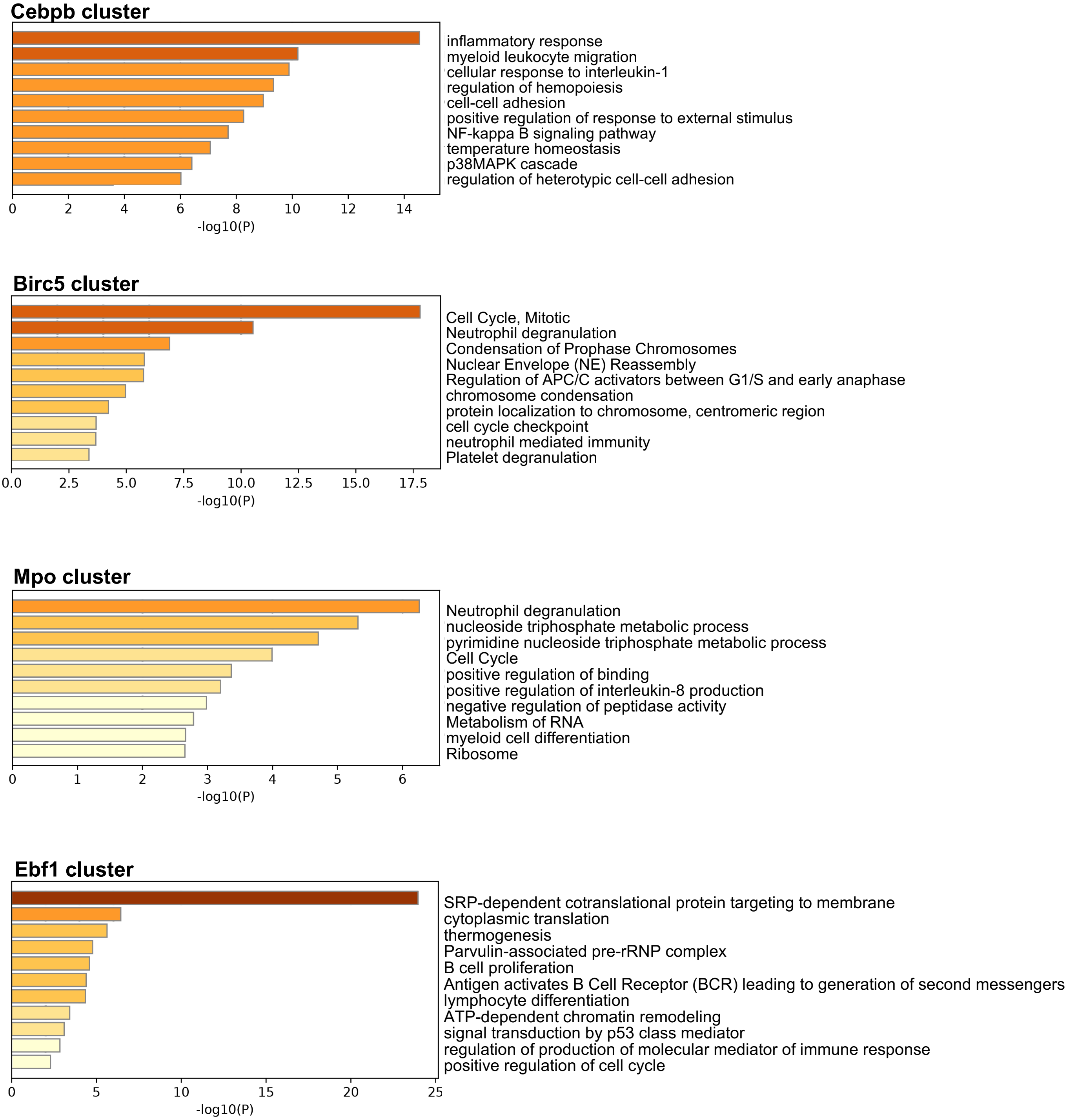

Supplementary Fig. 3 Functional enrichment analysis for the remaining clusters in the steady state.
(A)The top 60-65 markers (adj. p-value < 0.05) per cluster were used to identify the functional enrichment categories using Metascape. Pathway enrichment is expressed as the -log10(P) adjusted for multiple comparison.

Supplementary Figure 4

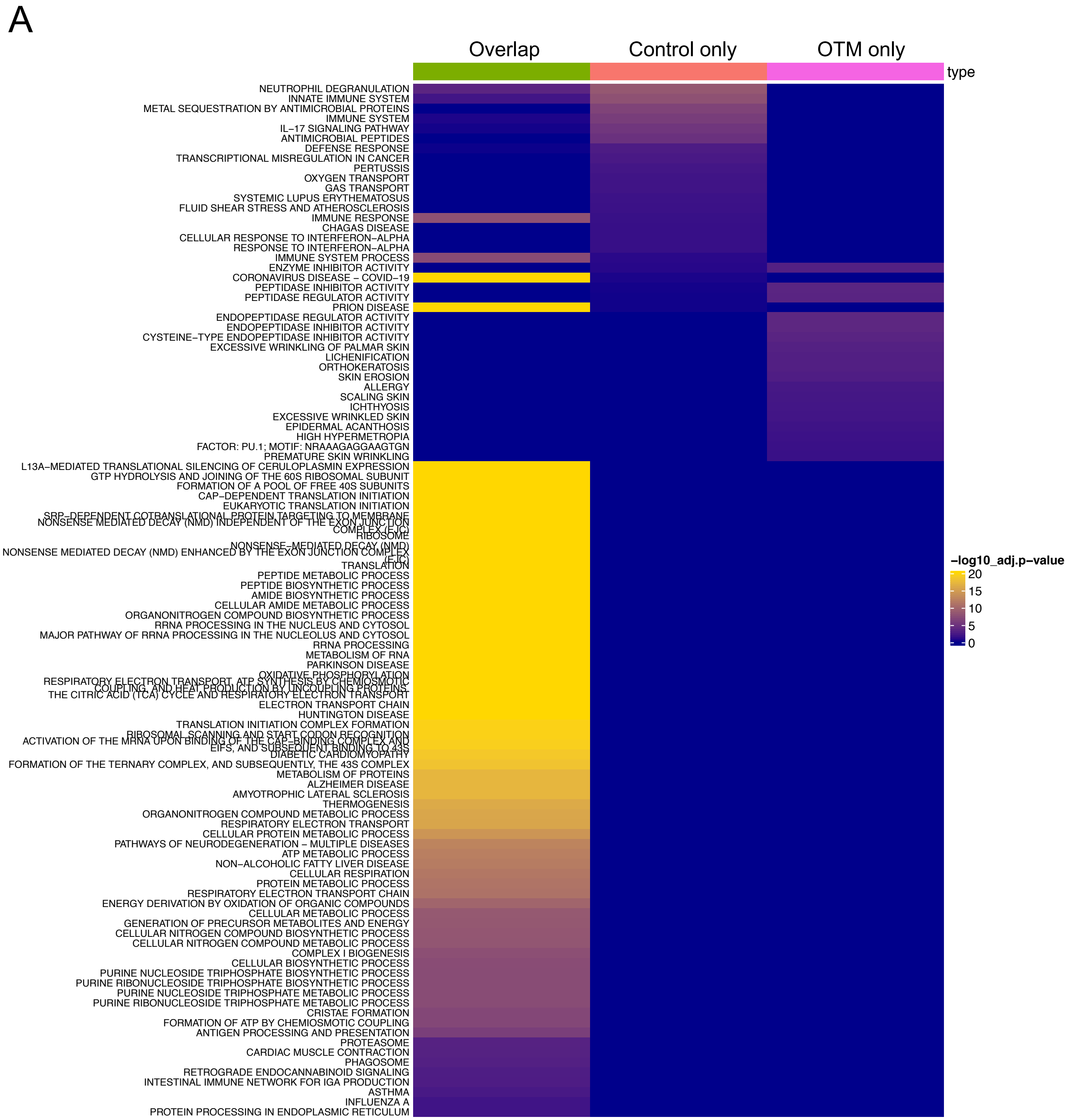

Supplementary Fig. 4 Heatmap of pathway analysis.

(A) Heatmap of pathway analysis (gProfiler) of differentially expressed genes in control only, OTM only, and overlapping populations.

Supplementary Figure 5

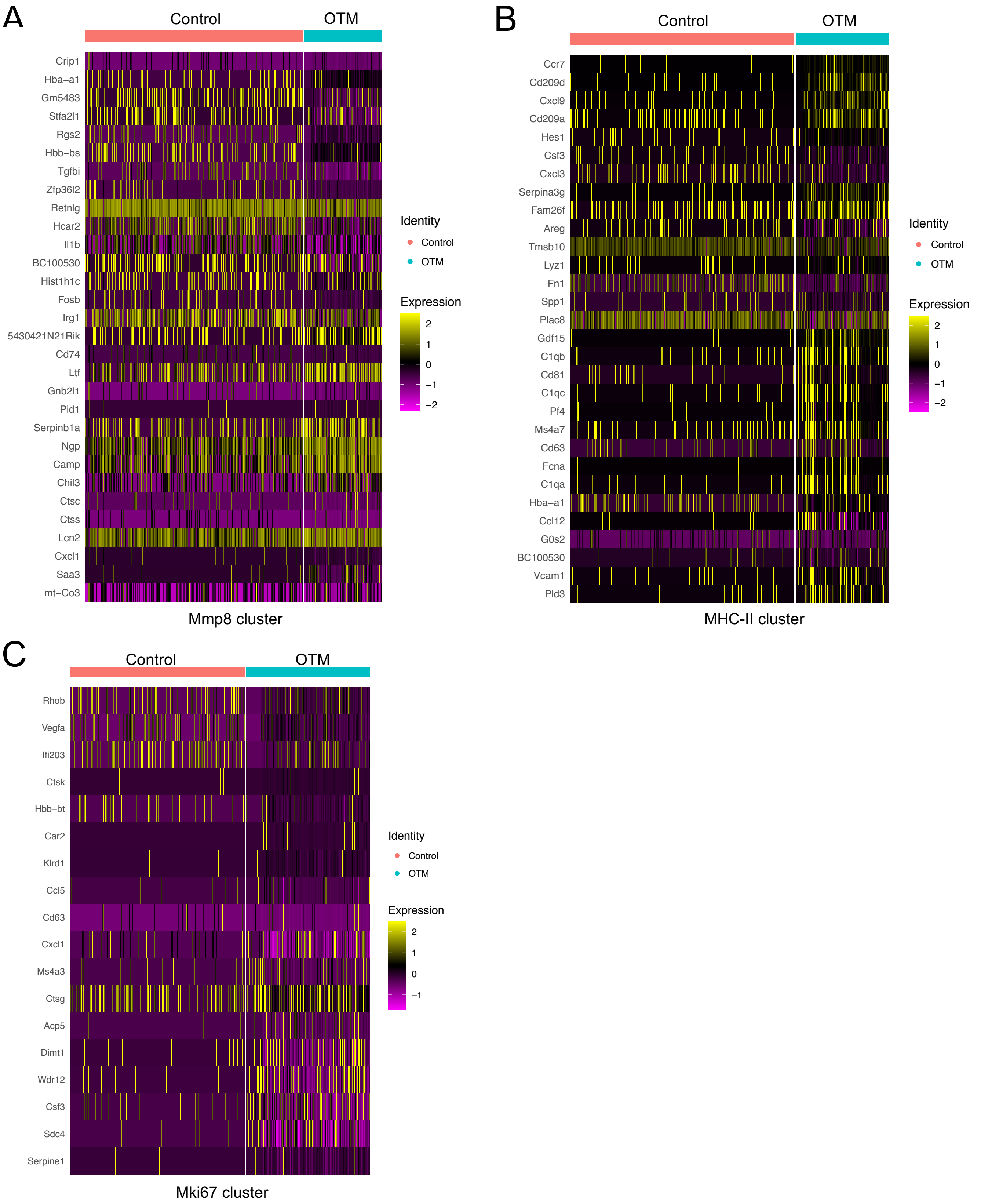

Supplementary Fig. 5 Heatmap of gene differentially expressed genes in common clusters between two groups. (A-C) Differentially expressed genes in *Mmp8*, *MHC-II*, *Mki67* cluster between control and OTM.

Supplementary Figure 6

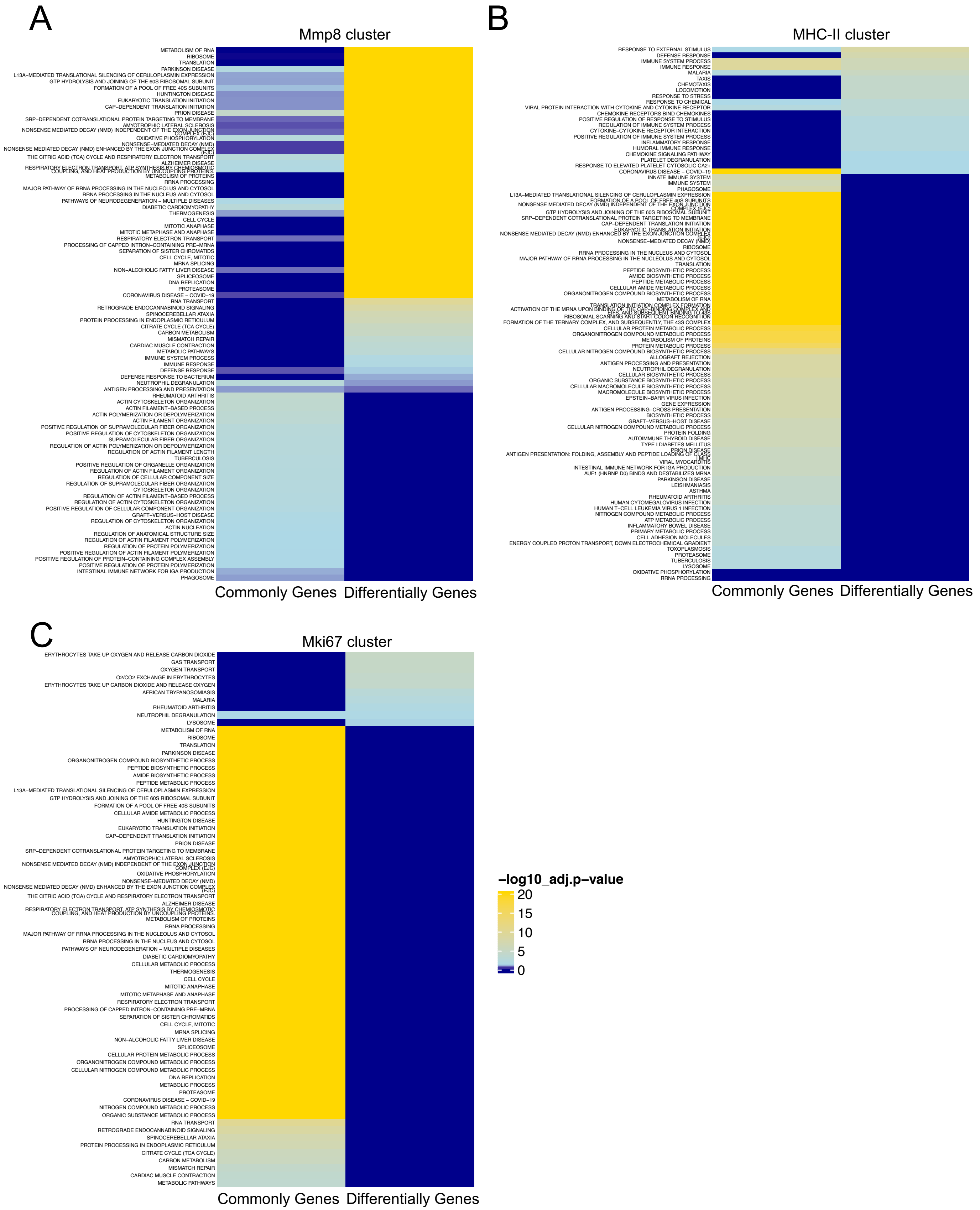

Supplementary Fig. 6 Heatmap of Pathway analysis in common clusters
(A-C) Pathway analysis (gProfiler) of commonly and differentially expressed genes in *Mmp8*, *MHC-II*, *Mki67* cluster.

Supplementary Figure 7

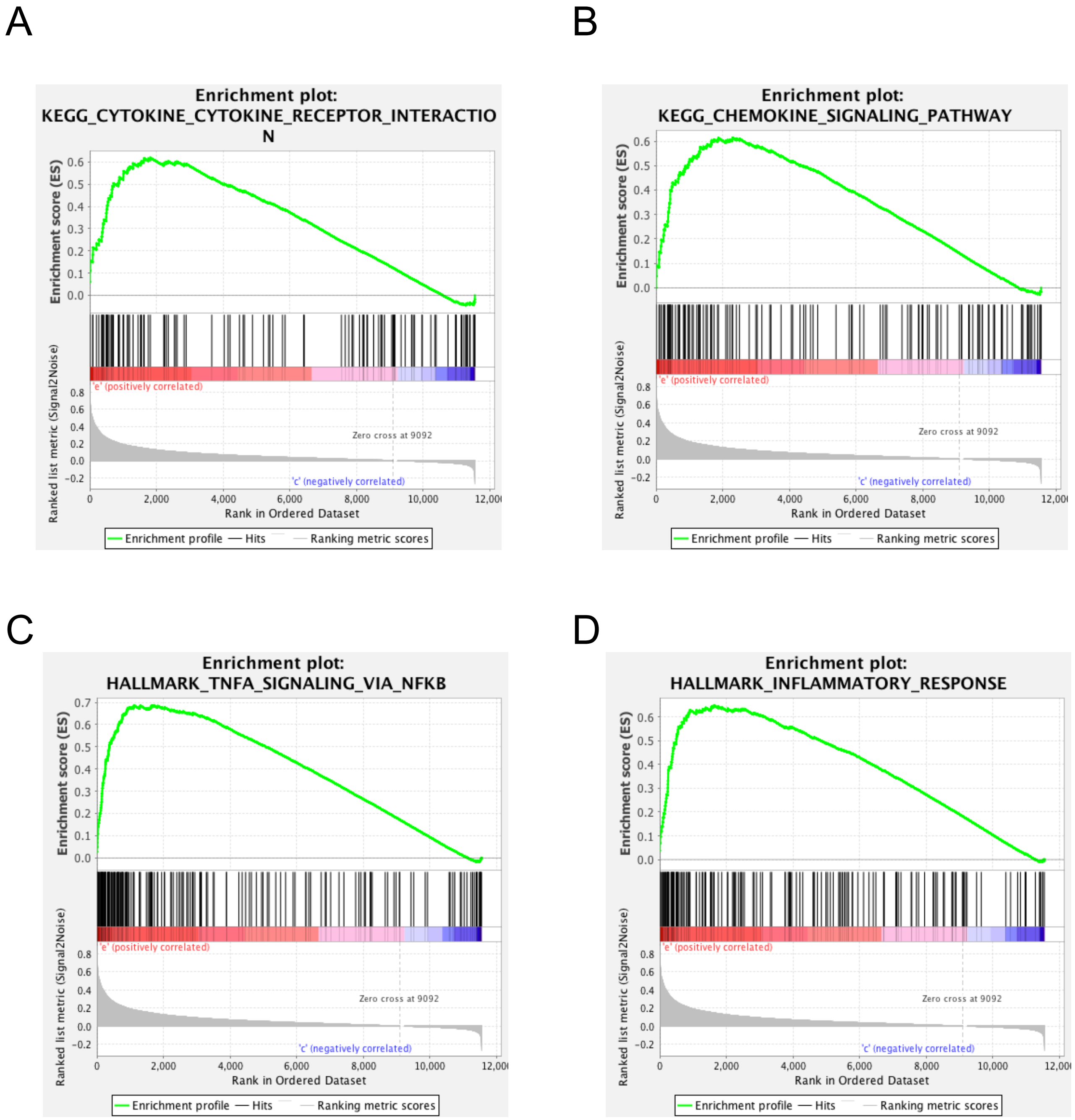

Supplementary Fig. 7 GSEA (gene set enrichment analysis) in *Ccr2* clusters between control and OTM.
(A-D) GSEA enrichment plots for representative signaling pathways upregulated in *Ccr2* cluster during OTM compared to normal state.
