# Supplementary material for: CCR2+ Macrophages Promote Orthodontic Tooth Movement and Alveolar Bone Remodeling
Source: Front Immunol. 2022 Feb 4;13:835986. doi: 10.3389/fimmu.2022.835986 (PMC8854866; doi:10.3389/fimmu.2022.835986)
Supplement: Supplementary file 2 [file Table_1.docx]

**Supplementary Table 1**

Control

| **Macrophage Cluster** | **proportion** | **Marker Genes** | **Major Function** |
| --- | --- | --- | --- |
| Cluster0= Ccr2 | 4.5% | *Ccr2, Ccl2, S100a4,* and *Fn1* | 'regulation of translation', 'myeloid cell homeostasis', 'cell killing', and 'cellular response to interleukin-1' |
| Cluster1= Mmp8 | 24.3% | *Retnlg, Mmp8* and *Mmp9* | 'neutrophil degranulation', 'leukocyte migration', and 'inflammatory response' |
| Cluster2= Ltf | 8.7% | *Ltf, Camp, Spp1,* and *Lcn2* | ‘neutrophil degranulation’, ‘leukocyte migration’, inflammatory response’ |
| Cluster3= Isg15 | 2.0% | *Isg15, Slfn4, Slfn1,* and *Ifit3* | regulation of defense response', 'defense response to virus', and 'response to interferon-beta' |
| Cluster4= Cebpb | 5.4% | *Il1b, Csf3r, Egr1, Ccrl2,* and *Cebpb* | 'inflammatory response', 'myeloid leukocyte migration', and 'response to IL-1' |
| Cluster5= MHC-II | 9.9% | *H2-Aa, Cd74, H2-Ab1, H2-Eb1,* and *H2-DMb1* | ‘antigen processing and presentation' and 'inflammatory response' |
| Cluster6= Fabp4 | 13.3% | *Fabp4, Apoe, Fcgr4, and Ace* | ‘fat cell differentiation', 'inflammatory response', and 'tissue remodeling' |
| Cluster7= Birc5 | 7.8% | *Ube2c, Stmn1, Birc5, Ltf*, and *Serpinb1a* | ‘cell cycle (mitosis)’ and ‘DNA packaging’ |
| Cluster8= Mik67 | 4.5% | *Stmn1, Ube2c, Birc5, Tubb5,* and *Top2a* | ‘cell cycle’, ‘response to DNA damage stimulus’ |
| Cluster9= Mpo | 15.3% | *Elane, Mpo, Prtn3, Ctsg and Nkg7* | ‘nucleoside triphosphate metabolism' and 'Interleukin-8 production' |
| Cluster10= Trem2 | 2.3% | *C1qa, Trem2, Pf4, Vcam-1 and Atp6vod2* | ‘osteoclasts differentiation’ |
| Cluster11= Ebf1 | 2.2% | *Ebf1, Cd79a, Cd79b* | ‘B cell proliferation' and 'lymphocyte differentiation' |

OTM

| **Macrophage Cluster** | **proportion** | **Marker Genes** | **Major Function** |
| --- | --- | --- | --- |
| Cluster 0= Ccr2 | 25.3% | *Ccr2, Ccl9* and *Fn1* | 'regulation of translation', 'myeloid cell homeostasis', 'cell killing', and 'cellular response to interleukin-1' |
| Cluster 1= Mmp8 | 16.7% | *Mmp9, Mmp8, Ly6g, Ltf,* and *Spp1* | ‘inflammatory response’ and ‘leukocyte migration’ |
| Cluster 2= Ccrl2 | 10.8% | *Ccrl2, Cxcr2, Cs43r,* and *Egr1* | ‘inflammatory response', ‘cytoplasmic translation’ and ‘bone resorption’ |
| Cluster 3= Elane | 9.2% | *Hmgn2, Ube2c, Tubb5, Ltf* and *Camp* | ‘cell cycle’, ‘DNA replication’, and ‘nucleotide metabolic process’ |
| Cluster 4= Ifitm1 | 9.4% | *Stfa2,* *Gm5483* *Asprv1, Ifitm1,* and *Mmp9* | ’classic inflammatory pathways, including inflammatory response and leukocyte migration |
| Cluster 5= Fcgr4 | 5% | *Cd83, Rgs1, Fcgr4* and *Apoe* | ‘response to external stimulus’ and ‘antigen processing and presentation’ |
| Cluster 6= MHC-II | 4.9% | *Cd74, H2-Aa, H2-Ab1,* and *H2-Eb1* | ‘antigen processing and presentation’ |
| Cluster 7= Mki67 | 18.6% | *Lgals1, Tmsb10, Tubb5,* and *Tubb1b* | ‘cell response to stimulus', 'proliferation-related', 'translational-ribosomal pathways' |

**Supplementary Table 2**

| **Gene** | **Forward primer (5’-3’)** | **Reverse primer (5’-3’)** |
| --- | --- | --- |
| *Gapdh* | AGGTCGGTGTGAACGGATTTG | TGTAGACCATGTAGTTGAGGTCA |
| *Il1β* | TCCAGGATGAGGACATGAGCAC | GAACGTCACACACCAGCAGGTTA |
| *Il6* | CCACTTCACAAGTCGGAGGCTTA | GCAAGTGCATCATCGTTGTTCATAC |
| *Tnfα* | GTTCTATGGCCCAGACCCTCAC | GGCACCACTAGTTGGTTGTCTTTG |
| *Il10* | GCCCTTTGCTATGGTGTCCTTTC | TCCCTGGTTTCTCTTCCCAAGAC |
| *Nos2* | CAGCTGGGCTGTACAAACCTT | ATGTGATGTTTGCTTCGGACA |
| *Chil3* | TACTCACTTCCACAGGAGCAGG | CTCCAGTGTAGCCATCCTTAGG |
| *Cd206* | CTCTGTTCAGCTATTGGACGC | CGGAATTTCTGGGATTCAGCTTC |
| *Ccr2* | TAGTTGCCCTGTATCTCCGC | TGGGGAAATGCGTCCTTGTT |
